# Supplementary material for: Inhibition of calcium-triggered secretion by hydrocarbon-stapled peptides
Source: Nature. 2022 Mar 23;603(7903):949–56. doi: 10.1038/s41586-022-04543-1 (PMC8967716; doi:10.1038/s41586-022-04543-1)
Supplement: Supplementary file 1 — Supplementary Figs 1–9. [file 41586_2022_4543_MOESM1_ESM.pdf]

nature portfolio

https://doi.org/10.1038/s41586-022-04543-1

Supplementary information

Inhibition of calcium-triggered secretion by hydrocarbon-stapled peptides

In the format provided by the authors and unedited

Table of Contents

Supplementary Figure 1. a, Annotated dot blots used to obtain the data shown in Fig.4 f-h. **b**, Annotated raw blots used to obtain the data plotted in Extended Data Fig. 8c-d.

Supplementary Figure 2. Quality control data for the SP9 peptide provided by Vivitide Inc.

Supplementary Figure 3. Quality control data for the TAT-SP9-Cy3 peptide provided by Vivitide Inc.

Supplementary Figure 4. Quality control data for the PEN-SP9-Cy3 peptide provided by Vivitid Inc.

Supplementary Figure 5. Quality control data for the PEN-P9-Cy3 peptide provided by Vivitide Inc.

Supplementary Figure 6. Quality control data for the TAT-P9-Cy3 peptide provided by Vivitide Inc.

Supplementary Figure 7. Quality control data for the SP9-Cy3 peptide provided by Vivitide Inc.

Supplementary Figure 8. Quality control data for the P0 peptide provided by Vivitide Inc.

Supplementary Figure 9. 1D and 2D NMR experiments for SP9.

Supplementary Table 1. Data summary table for the mouse airway mucin secretion and mucus occlusion experiments.

Supplementary Table 2. Data summary table for the single vesicle fusion experiments.

Supplementary Table 3. Donor subject sex, age and smoking status for HAEC experiments.

Supplementary Video S1. Video showing rocking views of the end points of five independent 1-µsec simulations of SP9 : Syt1-C2B. The corresponding coordinates are available as source data file SP9_simulations.pdb.

Supplementary Video S2. Video showing rocking views of the end points of five independent 1-µsec simulations of P9 : Syt1-C2B. The corresponding coordinates are available as source data file P9_simulations.pdb.

a Raw blots for figure 4 f-h

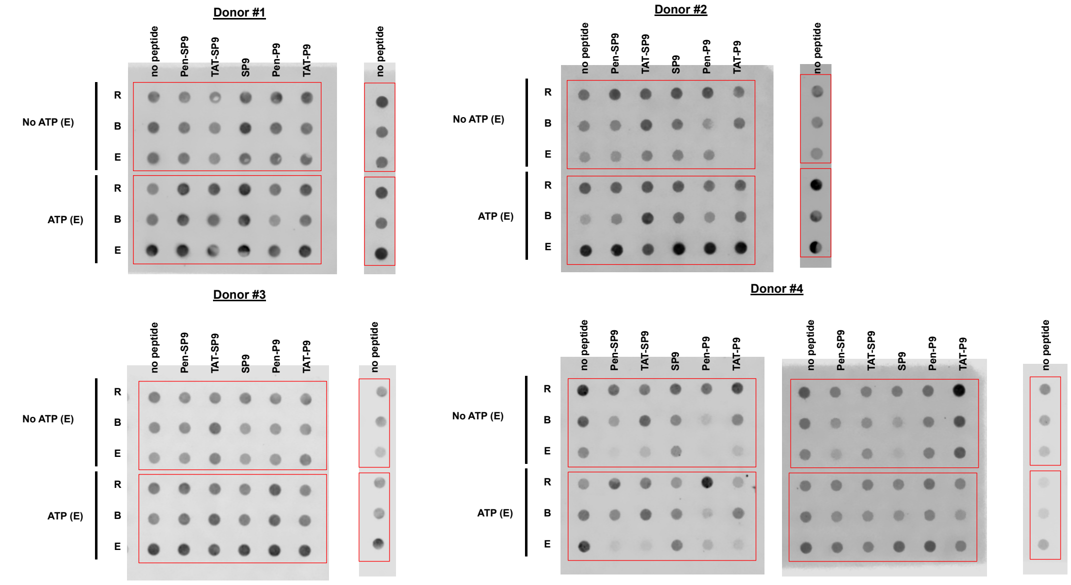

b Raw blots for Extended Data figure 8 c-d

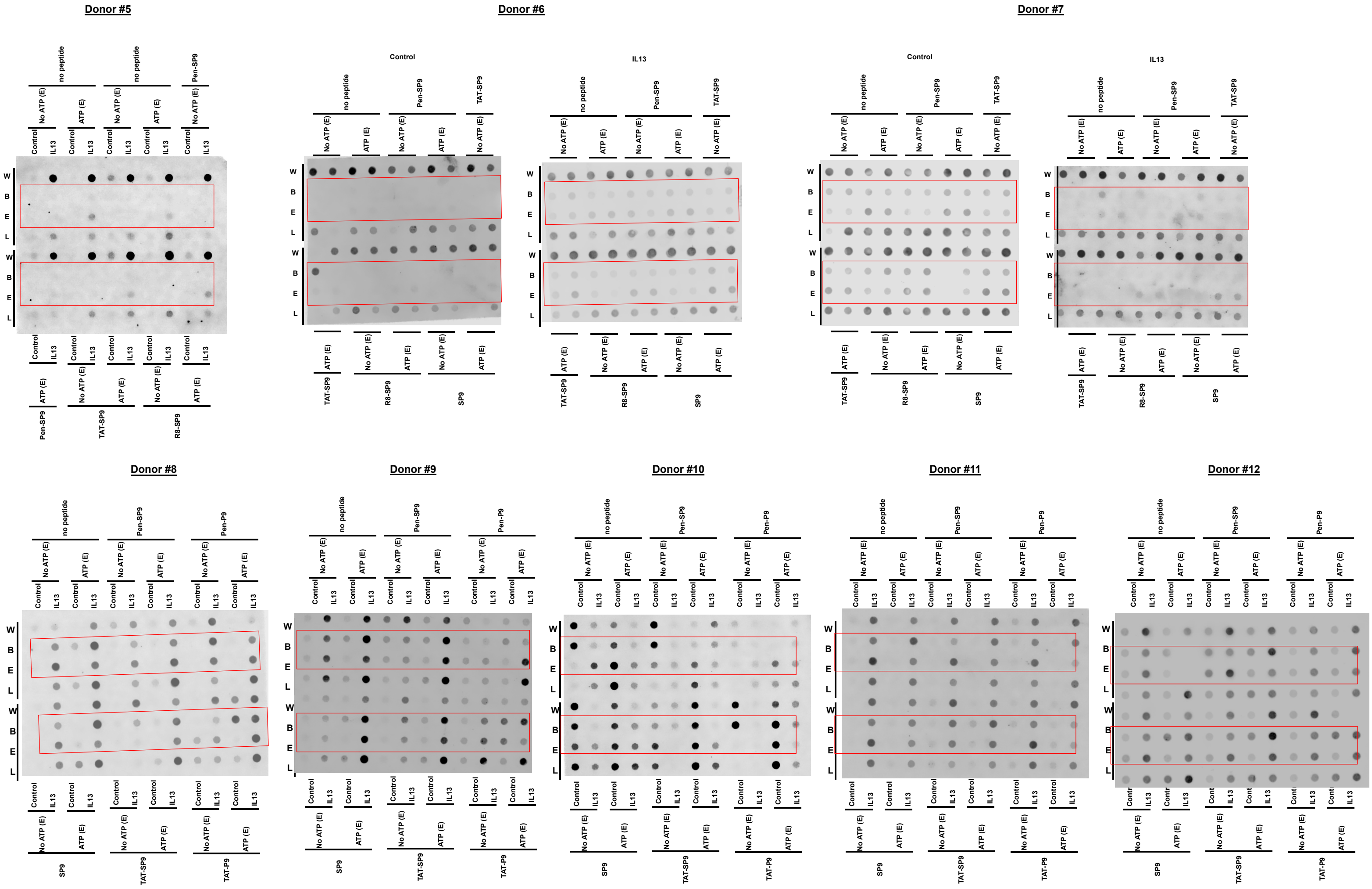

new england peptide™

Certificate of Analysis

**Sequence:** [Stp(3,7;10,14)]Ac-EE(S5)KDA(S5)IR(S5)LVM(S5)DEQC-amide

**Peptide Name:**	**Date:** January 7, 2019

**Order#:** P620387	**Lot#:** LB6578	**Amount:** 10.5mg

Quality Control Specifications:

QC Test	QC Specifications	Results
Purity by HPLC	>**95%** by percent area	**Pass**
Mass Identification by Mass Spectral Analysis	Calculated Mass within 0.1% of Molecular Weight: **2222**	**Pass**
Concentration/ Net Peptide	Amino Acid Analysis (AAA) determining original concentration/net peptide content.	N/A

Product: Research Grade Custom Peptide containing traces of Trifluoroacetate (TFA) salts.

Formulation:
Final concentration: N/A
Final form: Dry

Stability and Conditions: Refer to the Quality Control Detail Information on our website at www.newenglandpeptide.com/support/quality-control-information . As always, NEP has individual batch records stored electronically for each peptide that includes traceable lot numbers of raw materials used during synthesis. Should you require this information, email sales@newenglandpeptide.com with your peptide lot number.

Notes (if applicable):

PD
Approval/Initials

For Science...From Science.

New England Peptide Inc., 65 Zub Lane, Gardner, MA 01440 ■ **Phone** 888-343-5974 ■ **Fax** 978-630-0021

www.NewEnglandPeptide.com

Peptide QC Report LB6578 89-97

Analysis Name	D:\Data\LB6578 89-97_210010_P1-B-3_01_10382.D
Sample Name	LB6578 89-97
Method	APRIL20171.2mLperMIN_NEPOAHIGH_10382.m
Instrument	amaZon SL

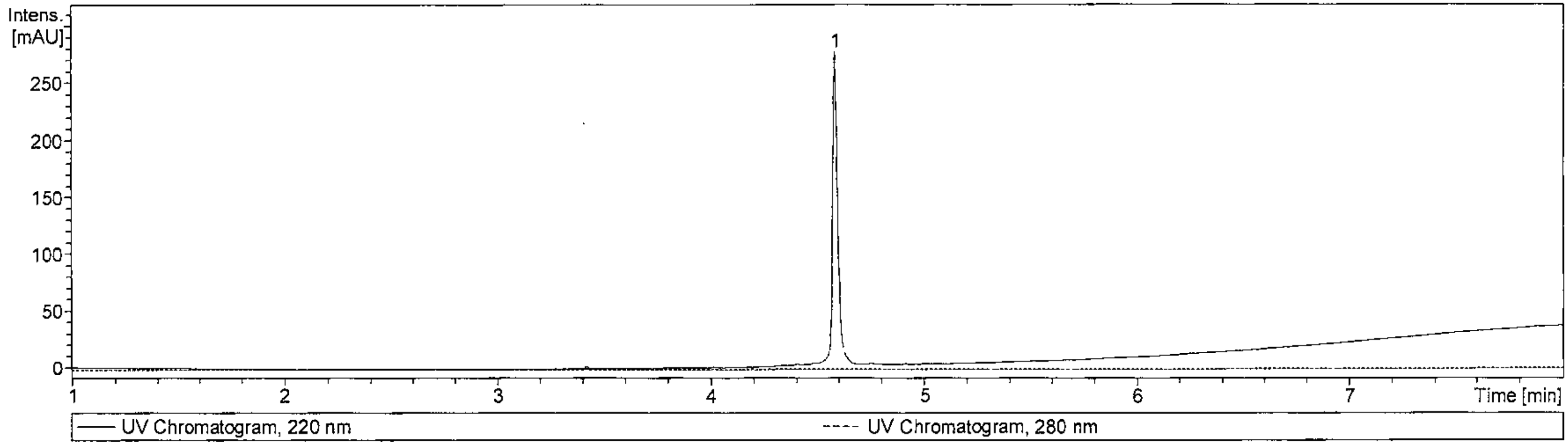

Target Mass	Meas. Mass	Expec. Mass	Delt. Mr [Da]	Intensity	Area	Area Fraction [%]
Cmpd 1; 4.58 min; Pep Mr: 2221.41	2221.41	2222.00	-0.59	276	469	100.0

#	RT [min]	Area	Area Frac. %
1	4.58	468.51	100.00

Peptide QC Report LB6578 89-97

Cmpd 1; 4.58 min; Pep Mr: 2221.41

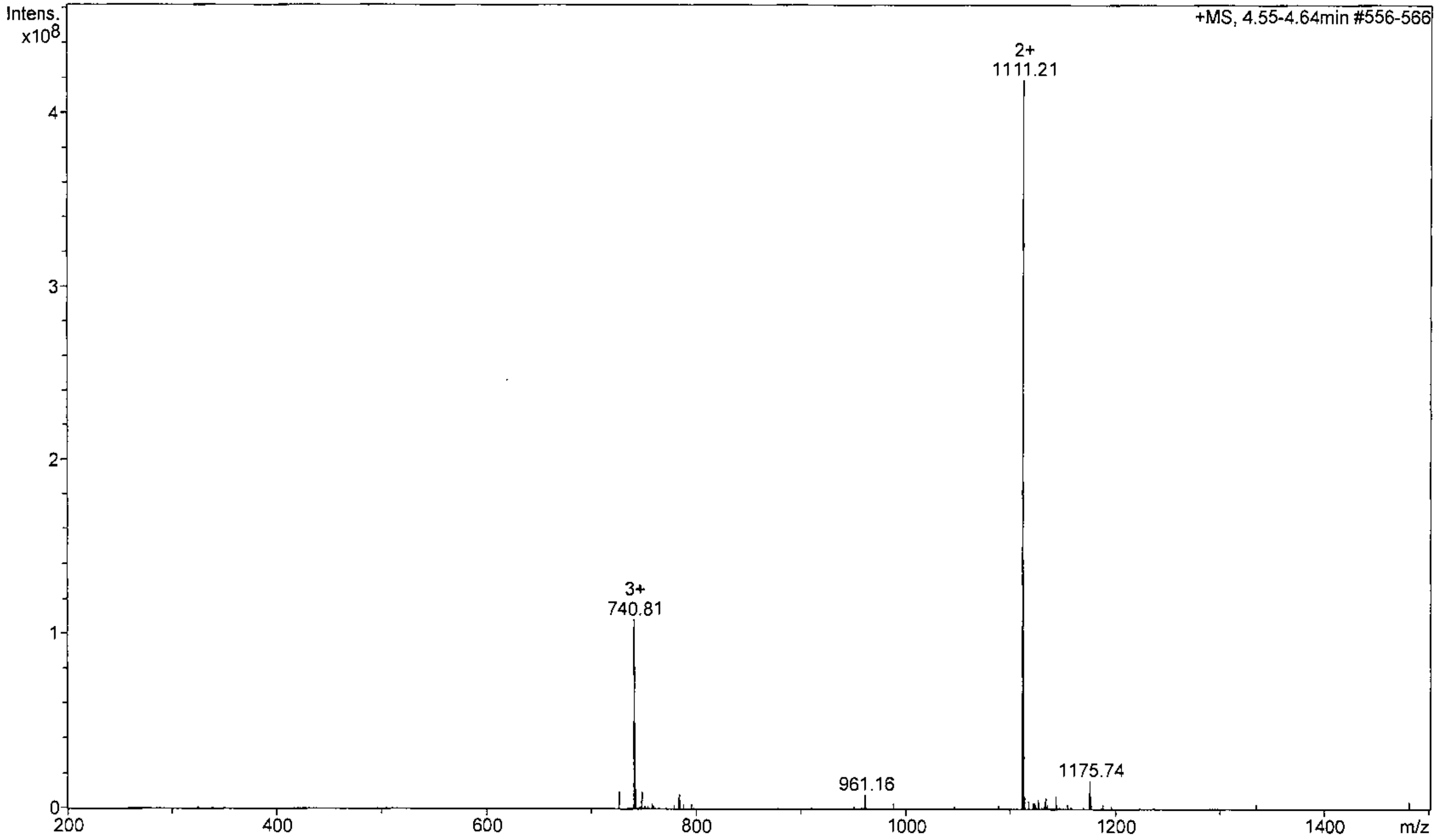

new england peptide™

Certificate of Analysis

**Sequence:** [Stp(16,20)][Stp(23,27)]H2N-GRKKRRQRRRPPQEE(S5)KDA(S5)IR(S5)LVM(S5)DEQ(C/Cy3Mal)-amide		
**Peptide Name:** TAT-SP9-Cy3		**Date:** May 8, 2020
**Order#:** P626925	**Lot#:** BU01353-3	**Amount:** 5.1mg

Quality Control Specifications:

QC Test	QC Specifications	Results
Purity by HPLC	**>95%** by percent area	**Pass**
Mass Identification by Mass Spectral Analysis	Calculated Mass within 0.1% of Molecular Weight: **4461.0**	**Pass**
Concentration/ Net Peptide	Amino Acid Analysis (AAA) determining original concentration/net peptide content.	**N/A**

Product: Research Grade Custom Peptide containing traces of Trifluoroacetate (TFA) salts.

Formulation:
Final concentration: N/A
Final form: Dry

Stability and Conditions: Refer to the Quality Control Detail Information on our website at www.newenglandpeptide.com/support/quality-control-information . As always, NEP has individual batch records stored electronically for each peptide that includes traceable lot numbers of raw materials used during synthesis. Should you require this information, email sales@newenglandpeptide.com with your peptide lot number.

Notes (if applicable): Peptide is light sensitive. A cis-trans isomerization of the Pro-Pro bond can occur causing a shoulder, wide peak, or split peak on the HPLC trace.

CR

Approval/Initials

Peptide QC Report BU01353

Analysis Name	D:\Data\BU01353_267370_P1-C-4_01_141129.D
Sample Name	BU01353
Method	APRIL20171.2mLperMIN_NEPOAHIGH_141129.m
Instrument	amaZon SL

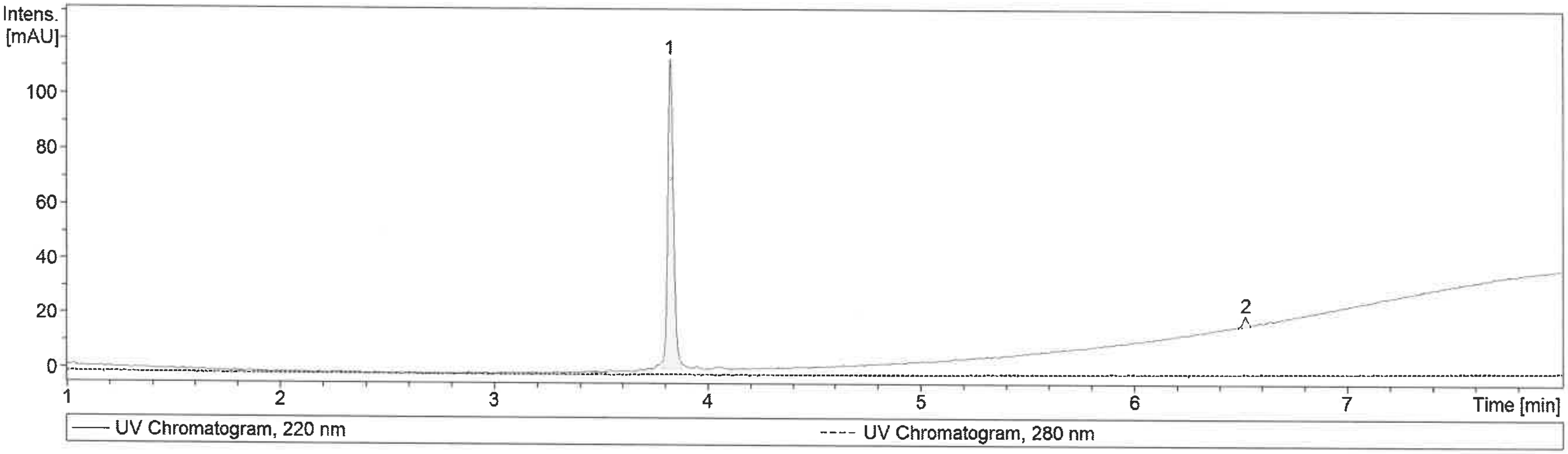

Target Mass	Meas. Mass	Expec. Mass	Delt. Mr [Da]	Intensity	Area	Area Fraction [%]
Cmpd 1; 3.82 min; Pep Mr: 3879.80	3879.80	3879.30	0.50	113	209	97.3

#	RT [min]	Area	Area Frac. %
1	3.82	209.1621	97.32
2	6.52	5.7706	2.68

nep new england peptide

Acquisition method name	D:\Methods\flexControlMethods\Positive Peptides 500-3500 Sep_2018.par
Date of Acquisition	2020-01-10T22:49:37.187-05:00
Comment 2	BU01353
LaserPower = 100% - Laser beam attenuation	81.296
Number of shots	300
Current absolute file path of the spectrum	Z:\BEV\BU01353\0_G5\2\1SLin
Sample name (file name prefix)	BU01353\0_G5\2
Position	G5
positive voltage polarity	POS

Sample ID: BU01353 Molecular Weight: 3879.3

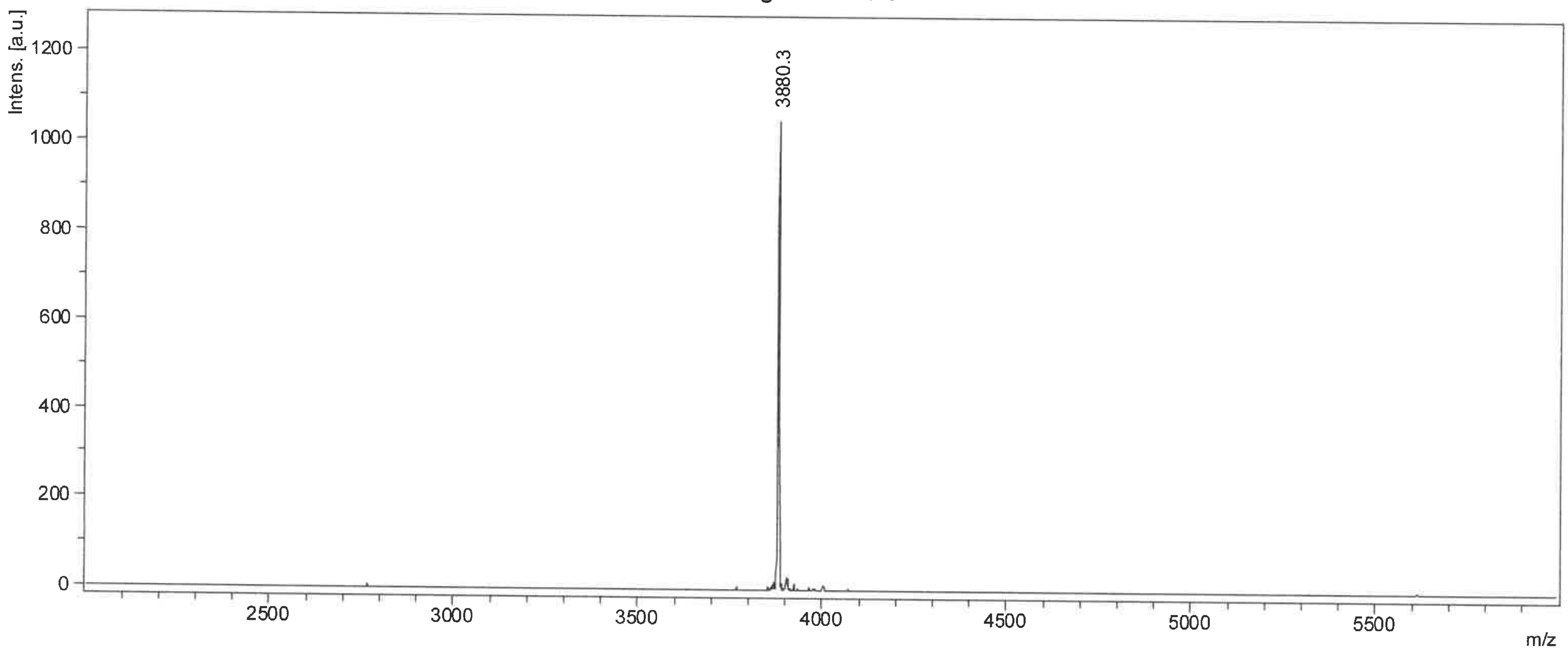

new england peptide™

Certificate of Analysis

Sequence: [Stp(19,23)][Stp(26,30)]H2N-RQIKIWFQNRRMKWKKEE(S5)KDA(S5)IR(S5)LVM(S5)DEQ(C/Cy3Mal)-amide

**Peptide Name:** PEN - SPq - CY3		**Date:** August 3, 2020
**Order#:** P627037	**Lot#:** BU06012	**Amount:** 5.4mg

Quality Control Specifications:

QC Test	QC Specifications	Results
Purity by HPLC	**>90%** by percent area	**Pass**
Mass Identification by Mass Spectral Analysis	Calculated Mass within 0.1% of Molecular Weight: **4986.1**	**Pass**
Concentration/ Net Peptide	Amino Acid Analysis (AAA) determining original concentration/net peptide content.	**N/A**

Product: Research Grade Custom Peptide containing traces of Trifluoroacetate (TFA) salts.

Formulation:
Final concentration: N/A
Final form: Dry

Stability and Conditions: Refer to the Quality Control Detail Information on our website at www.newenglandpeptide.com/support/quality-control-information . As always, NEP has individual batch records stored electronically for each peptide that includes traceable lot numbers of raw materials used during synthesis. Should you require this information, email sales@newenglandpeptide.com with your peptide lot number.

Notes (if applicable): Peptide is light sensitive.

BAS

Approval/Initials

Peptide QC Report BU06012 32-52

Analysis Name	D:\Data\BU06012 32-52_301767_P1-E-3_01_52123.d
Sample Name	BU06012 32-52
Method	APRIL20171.2mLperMIN_NEPOAHIGH_52123.m
Instrument	amaZon SL

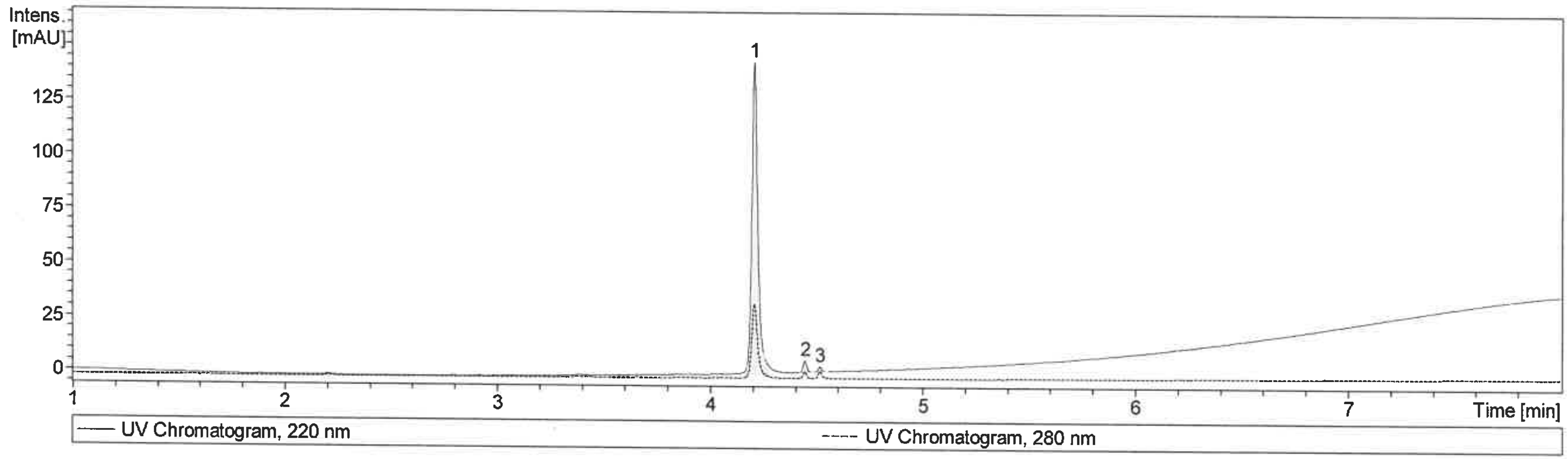

Target Mass	Meas. Mass	Expec. Mass	Delt. Mr [Da]	Intensity	Area	Area Fraction [%]
Cmpd 1; 4.21 min; Pep Mr: 4986.18	4986.18	4986.10	0.08	143	280	96.7

#	RT [min]	Area	Area Frac. %
1	4.21	280.3262	96.69
2	4.45	6.6779	2.30
3	4.51	2.9193	1.01

Peptide QC Report BU06012 32-52

Cmpd 1; 4.21 min; Pep Mr: 4986.18

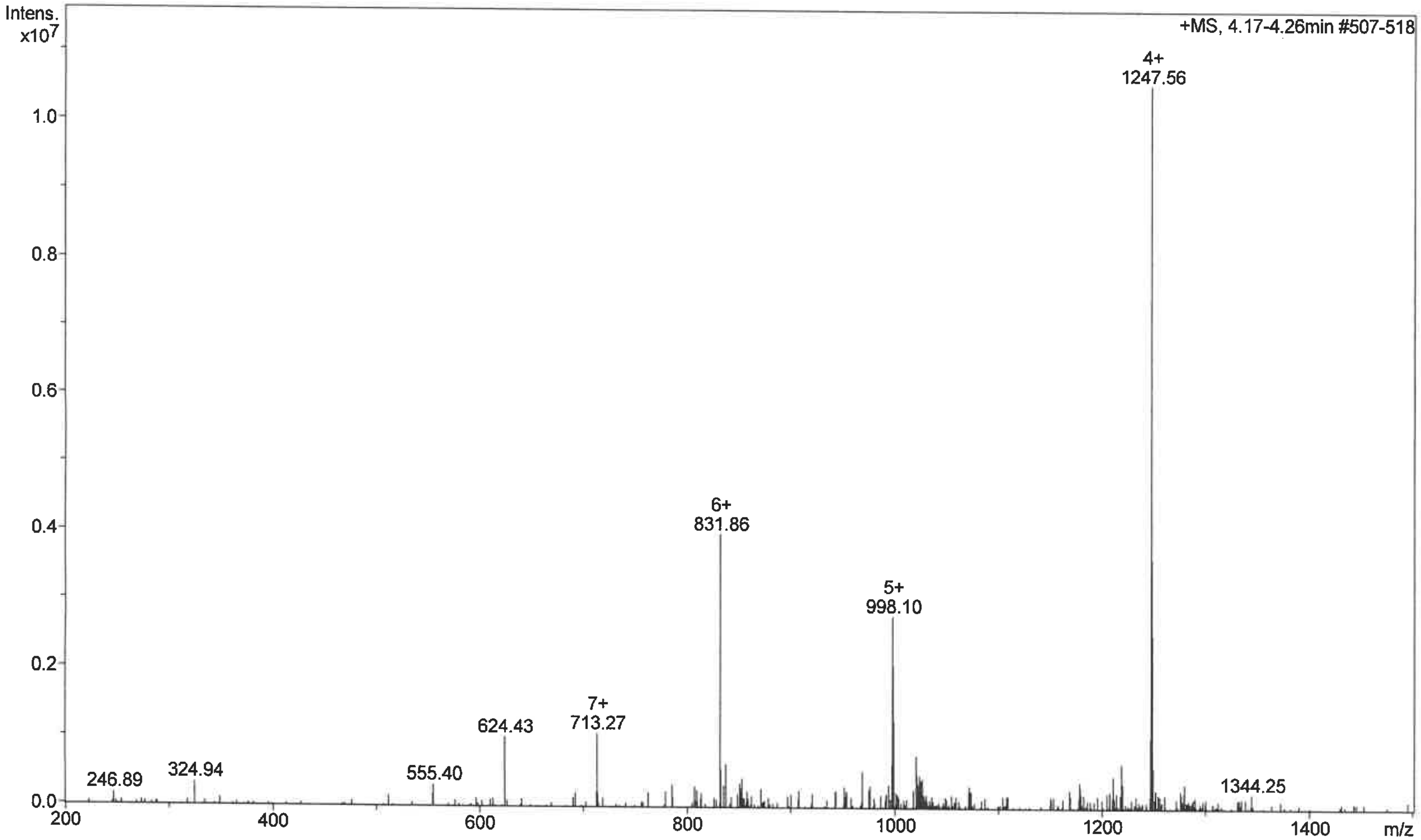

Certificate of Analysis

**Sequence:** H2N-RQIKIWFQNRRMKWKKEESKDAGIRTLVMLDEQ(C/Cy3Mal)-amide	
**Peptide Name:** Penetratin-P9-Cy3	**Date:** February 1, 2021

**Order#:** P629945	**Lot#:** BU08265	**Amount:** 5.2mg

Quality Control Specifications:

QC Test	QC Specifications	Results
Purity by HPLC	**>90%** by percent area	**Pass**
Mass Identification by Mass Spectral Analysis	Calculated Mass within 0.1% of Molecular Weight: **4844.8**	**Pass**
Concentration/ Net Peptide	Amino Acid Analysis (AAA) determining original concentration/net peptide content.	**N/A**

Product: Research Grade Custom Peptide containing traces of Trifluoroacetate (TFA) salts.

Formulation:
Final concentration: N/A
Final form: Dry

Stability and Conditions: Refer to the Quality Control Detail Information on our website at www.newenglandpeptide.com/support/quality-control-information . As always, NEP has individual batch records stored electronically for each peptide that includes traceable lot numbers of raw materials used during synthesis. Should you require this information, email sales@newenglandpeptide.com with your peptide lot number.

Notes (if applicable): Peptide is light sensitive.

UR

Approval/Initials

For Science...From Science.

New England Peptide Inc., 65 Zub Lane, Gardner, MA 01440 ▪ **Phone** 888-343-5974 ▪ **Fax** 978-630-0021

www.NewEnglandPeptide.com

new england peptide

Peptide QC Report BU08265 8-23

Analysis Name	D:\Data\NEP\BU08265 8-23_P1-C-9_1_2319.d
Sample Name	BU08265 8-23
Method	NEPLC_HIGH_2319.m
Instrument	amaZon SL 3

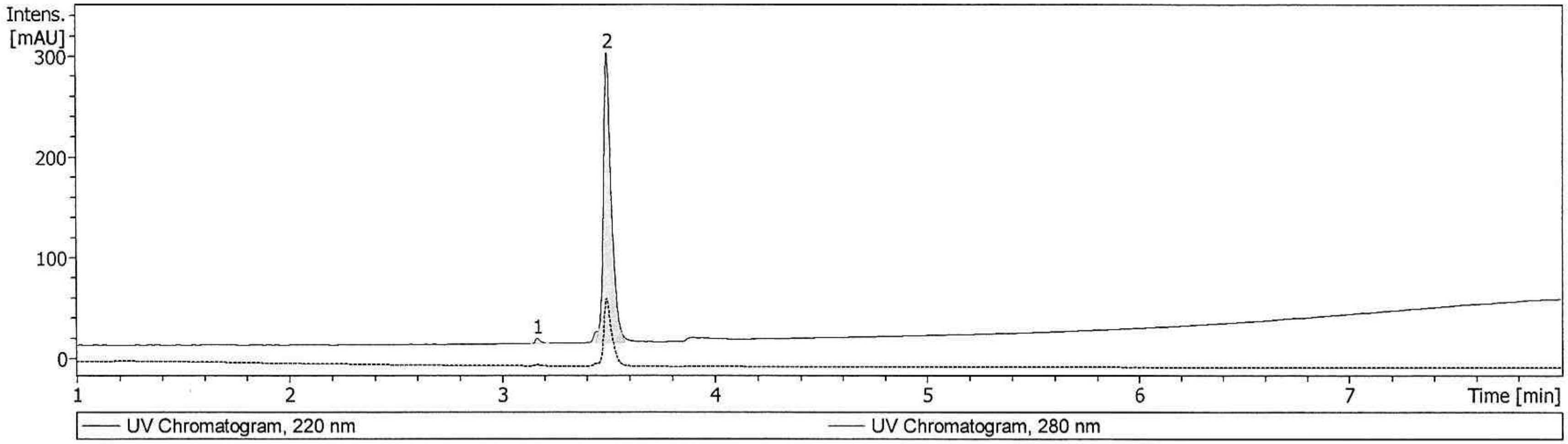

Target Mass	Meas. Mass	Expec. Mass	Delt. Mr [Da]	Intensity	Area	Area Fraction [%]
Cmpd 2; 3.49 min; Pep Mr: 4844.63	4844.63	4844.80	-0.17	303	742	98.7

#	RT [min]	Area	Area Frac. %
1	3.17	9.8363	1.31
2	3.49	742.3948	98.69

Peptide QC Report BU08265 8-23

Cmpd 2; 3.49 min; Pep Mr: 4844.63

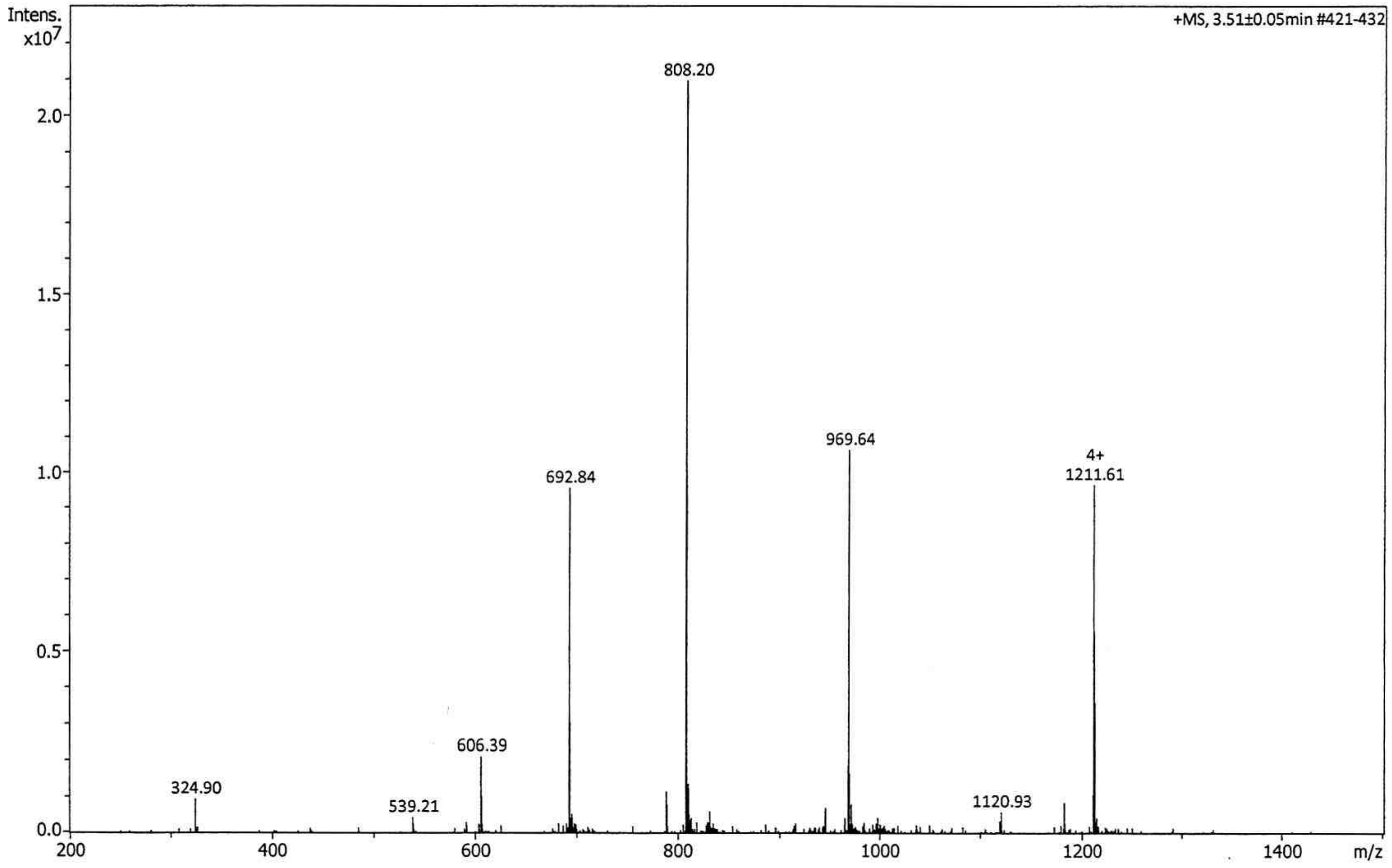

new england peptide™

Certificate of Analysis

TAT P9

**Sequence:** H2N-GRKKRRQRRRPPQEESKDAGIRTLVMLDEQ(C/Cy3Mal)-amide

**Peptide Name:** TAT-P9-Cy3	**Date:** October 8, 2019

**Order#:** P623777	**Lot#:** BU01237	**Amount:** 2.4mg

Quality Control Specifications:

QC Test	QC Specifications	Results
Purity by HPLC	**>95%** by percent area	**Pass**
Mass Identification by Mass Spectral Analysis	Calculated Mass within 0.1% of Molecular Weight: **4316**	**Pass**
Concentration/ Net Peptide	Amino Acid Analysis (AAA) determining original concentration/net peptide content.	**N/A**

Product: Research Grade Custom Peptide containing traces of Trifluoroacetate (TFA) salts.

Formulation:
Final concentration: N/A
Final form: Dry

Stability and Conditions: Refer to the Quality Control Detail Information on our website at www.newenglandpeptide.com/support/quality-control-information . As always, NEP has individual batch records stored electronically for each peptide that includes traceable lot numbers of raw materials used during synthesis. Should you require this information, email sales@newenglandpeptide.com with your peptide lot number.

Notes (if applicable): A cis-trans isomerization of the Pro-Pro bond can occur causing a shoulder, wide peak, or split peak on the HPLC trace. Peptide is light sensitive.

DW

Approval/Initials

Peptide QC Report BU01237 29-40

Analysis Name	D:\Data\BU01238 29-40_251905_P2-A-7_01_30537.d
Sample Name	BU01237 29-40
Method	APRIL20171.2mLperMIN_NEPO A_30537.m
Instrument	UPLC1

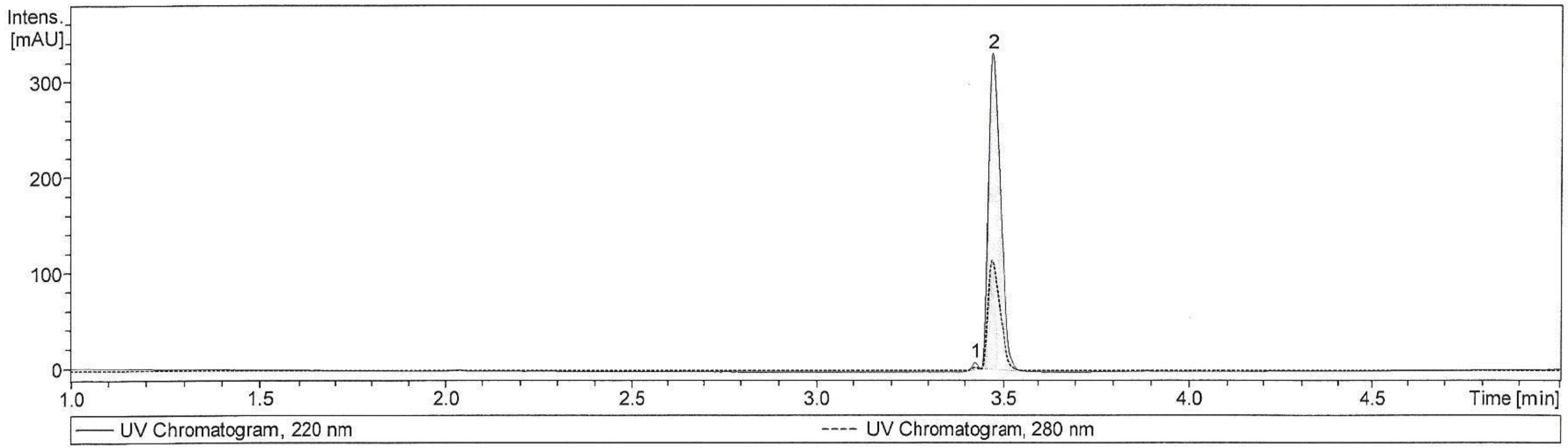

	Target Mass	Meas. Mass	Expec. Mass	Delt. Mr [Da]	Intensity	Area	Area Fraction [%]
Cmpd 2; 3.47 min; Pep Mr:	4316.06	4316.06	4316.00	0.06	328	705	98.9

#	RT [min]	Area	Area Frac. %
1	3.43	7.5006	1.05
2	3.47	704.6333	98.95

Peptide QC Report BU01237 29-40

Cmpd 2; 3.47 min; Pep Mr: 4316.06

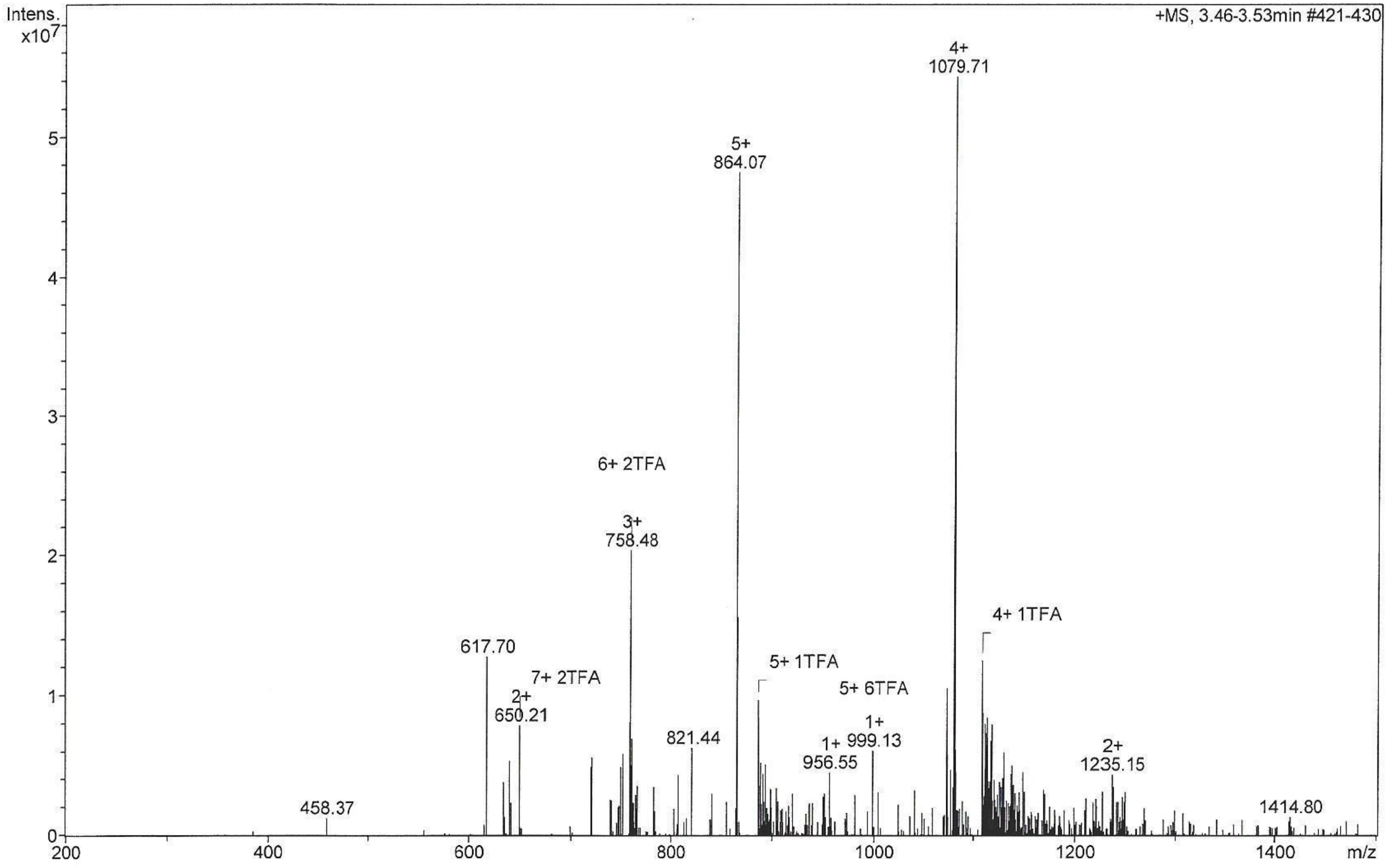

vivitide
Science for life

Certificate of Analysis

Sequence: [Stp(3,7)][Stp(10,14)]Ac-EE(S5)KDA(S5)IR(S5)LVM(S5)DEQ(C/Cy3Mal)-amide

**Peptide Name:**	**Date of Manufacture:** October 21, 2021

**Order #:** P634650	**Lot#:** BU11780-2	**Amount:** 1.5mg

Quality Control Specifications:

QC Test	QC Specifications	Results
Purity by HPLC	**>95%** by percent area	**Pass**
Mass Identification by Mass Spectral Analysis	Calculated Mass within +/-1 Da of average Molecular Weight: **2800.0**	**Pass**
Concentration / Net Peptide Content	Amino Acid Analysis (AAA) determining original concentration/net peptide content.	**N/A**
Salt Form		Trifluoroacetate (TFA)

Product: Research Grade Custom Peptide.

Formulation:
Final concentration: N/A
Final form: Dry

Stability and Conditions: Refer to the Quality Control Detail Information on our website at https://vivitide.com/pages/quality-control. As always, vivitide has individual batch records stored electronically for each peptide that includes traceable lot numbers of raw materials used during production. Should you require this information, email sales@vivitide.com with your peptide lot number.

Notes (if applicable):
Peptide is light sensitive.

BAS
Approved by:

65 Zub Lane • Gardner, MA 01440 • USA
P 1-978-630-0020 • 1-888-343-5974 • 1-978-630-0021
sales@vivitide.com

11621 Electron Drive • Louisville, KY 40207 • USA
P 1-502-266-8787 • 1-800-777-4779 • F 1-502-267-1329
sales@vivitide.com

vivitide
Science for life

Peptide QC Report BU11780-2 11-15

Analysis Name	D:\Data\NEP\BU11780-2 11-15_P1-B-8_1_17663.d
Sample Name	BU11780-2 11-15
Method	NEPLC_HIGH_17663.m
Instrument	amaZon SL 3

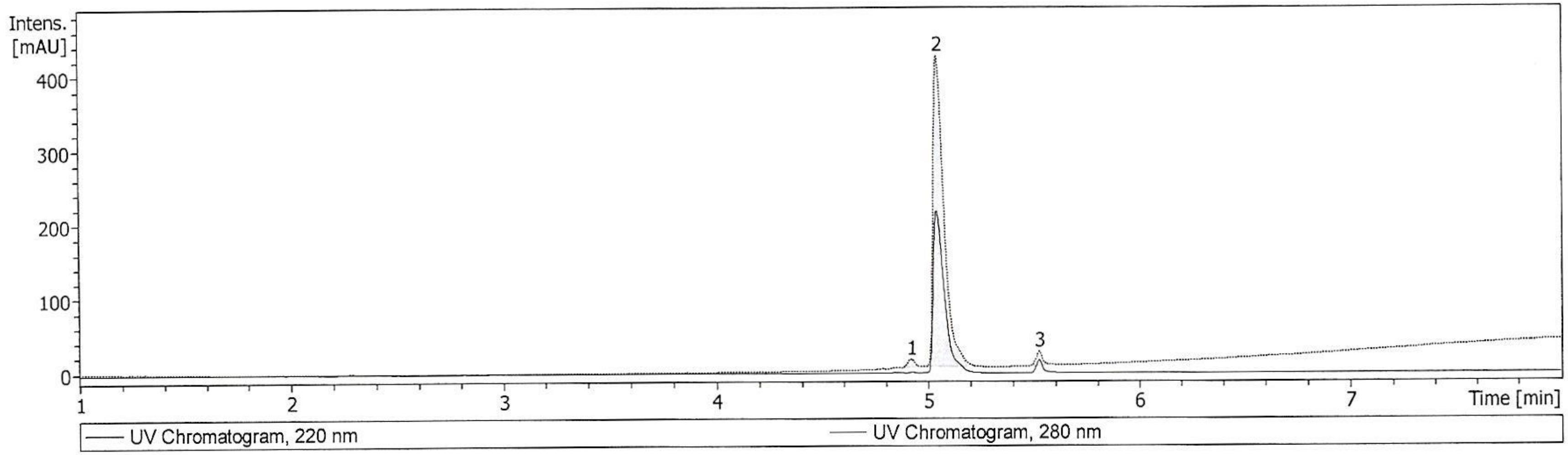

Target Mass	Meas. Mass	Expec. Mass	Delt. Mr [Da]	Intensity	Area	Area Fraction [%]
Cmpd 2; 5.04 min; Pep Mr: 2799.67	2799.67	2800.00	-0.33	425	1481	95.9

#	RT [min]	Area	Area Frac. %
1	4.92	24.923	1.61
2	5.04	1480.994	95.87
3	5.52	38.936	2.52

Peptide QC Report BU11780-2 11-15

Cmpd 2; 5.04 min; Pep Mr: 2799.67

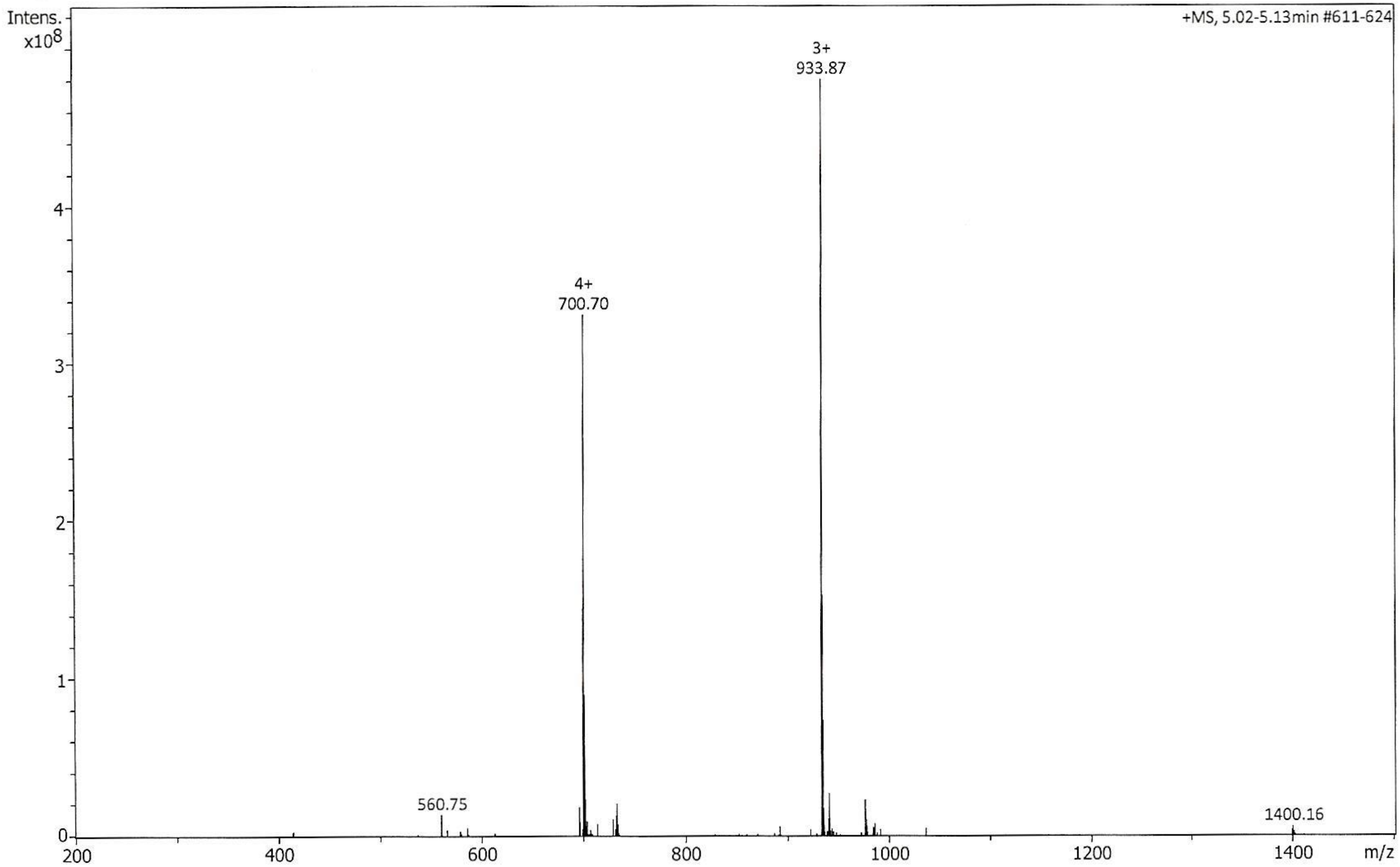

new england peptide™

Certificate of Analysis

**Sequence:** H2N-EESKDAGIRTLVMLDEQGEQLD-OH		
**Peptide Name:**		**Date:** 12/11/2017
**Order#:** P615100	**Lot#:** F2561	**Amount:** 10.0mg

Quality Control Specifications:

QC Test	QC Specifications	Results
Purity by HPLC	>95% by percent area	**Pass**
Mass Identification by Mass Spectral Analysis	Calculated Mass within 0.1% of Molecular Weight: **2477**	**Pass**
Concentration/ Net Peptide	Amino Acid Analysis (AAA) determining original concentration/net peptide content.	**N/A**

Product: Research Grade Custom Peptide containing traces of Acetate salts.

Formulation:
Final concentration: N/A
Final form: Dry

Stability and Conditions: Refer to the Quality Control Detail Information on our website at www.newenglandpeptide.com/support/quality-control-information . As always, NEP has individual batch records stored electronically for each peptide that includes traceable lot numbers of raw materials used during synthesis. Should you require this information, email sales@newenglandpeptide.com with your peptide lot number.

Notes (if applicable):

PD

Approval/Initials

For Science... From Science.

New England Peptide Inc., 65 Zub Lane, Gardner, MA 01440 ■ **Phone** 888-343-5974 ■ **Fax** 978-630-0021

www.NewEnglandPeptide.com

Peptide QC Report F2561 77-95

Analysis Name	D:\Data\F2561 77-95_160310_P1-E-3_01_80094.D
Sample Name	F2561 77-95
Method	APRIL20171.2mLperMIN_NEPO A_80094.m
Instrument	amaZon SL

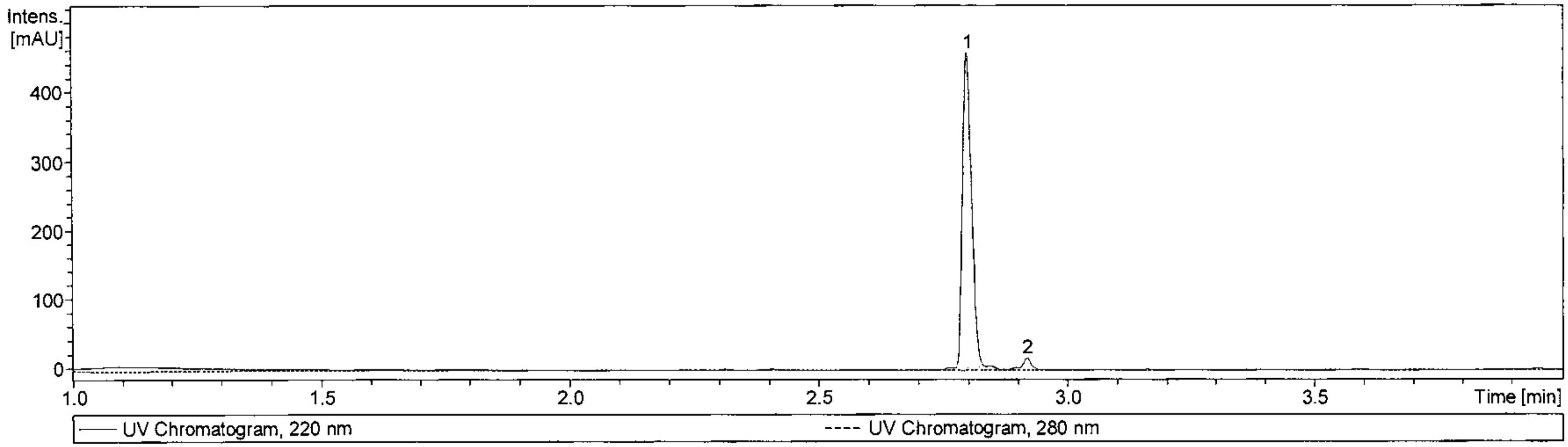

Target Mass	Meas. Mass	Expec. Mass	Delt. Mr [Da]	Intensity	Area	Area Fraction [%]
Cmpd 1; 2.80 min; Pep Mr: 2476.41	2476.41	2477.00	-0.59	454	581	96.6

#	RT [min]	Area	Area Frac. %
1	2.80	580.604	96.56
2	2.92	20.703	3.44

Cmpd 1; 2.80 min; Pep Mr: 2476.41

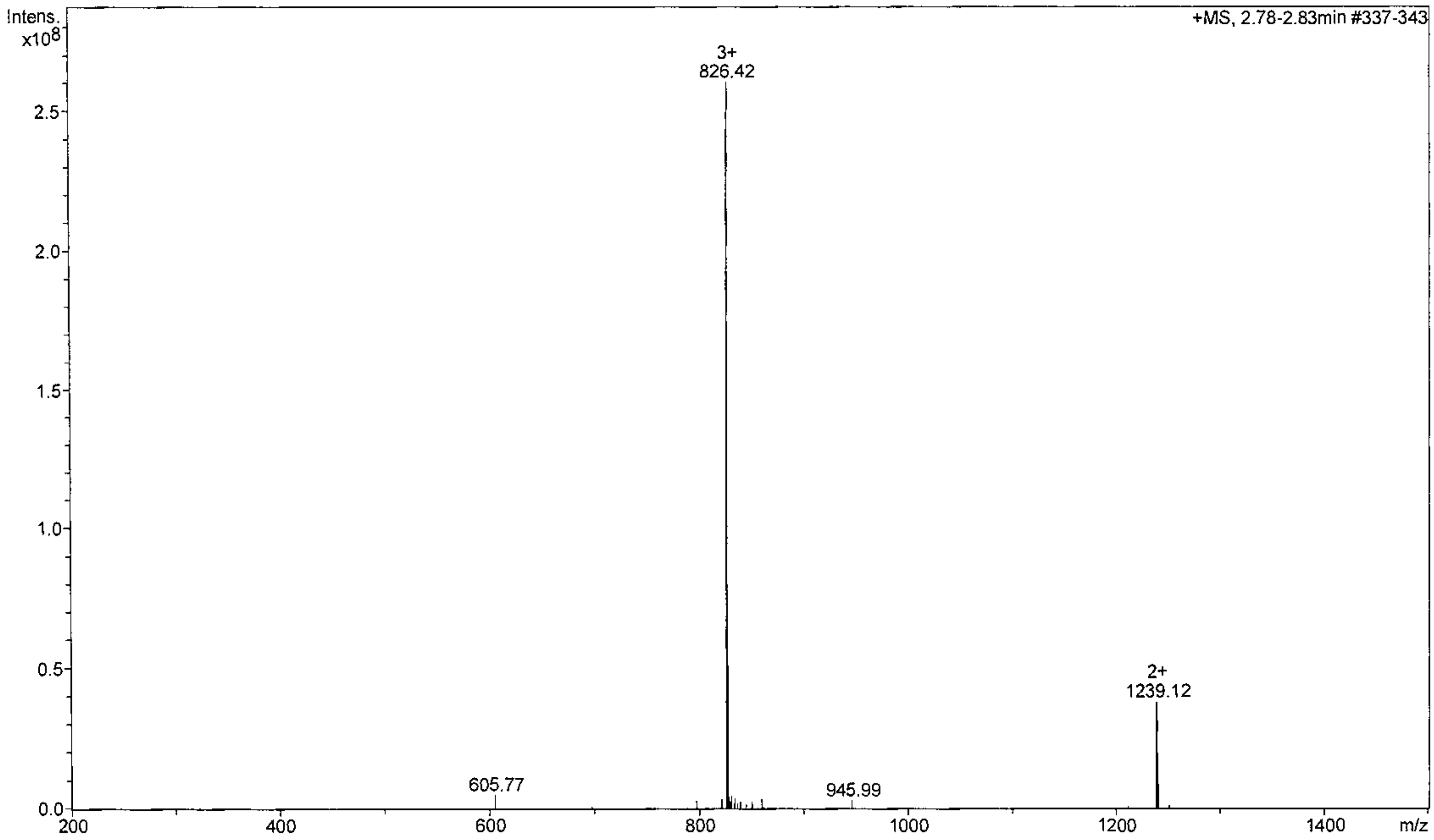

Supplementary Figure 9

NMR studies of SP9 (spectra are below)

NMR data were acquired on the peptide sample on a 600 MHz for H1 Varian Unity Inova NMR system with a 5 mm HCN room temperature probe. H1 1D, H1/C13 2D gHSQC, H1/C13 gHMBC, H1/H1 gCOSY, H1/H1 TOCSY with an 80 ms mixing time and H1/H1 ROESY with a 200 ms mixing time were acquired and analyzed with VNMRJ and MestreNova software.

The 1D data was integrated to analyze the number of downfield shifted H1 resonances, which would be only NH, NH2, and NH3 resonances in this peptide. The gHSQC was used to disperse the resonances by C13 chemical shift. The α H1 resonances can thus be counted as they are between 3.4 and 4.5 in H1 shift and 50 and 65 in C13 shift. The remainder of the side chain resonances are somewhat dispersed using gHSQC, but the overlap of those was insufficient to readily identify all of them.

Each amino acid has characteristic shifts for the side chain resonances as well as the α H1 and C13. To determine which α H1 and NH resonance belong to each amino acid the TOCSY and gCOSY were used to follow the NH to Hα out the side chain. The TOCSY connects all H1 resonances in the same spin system; the gCOSY connects to only the H1 resonances on adjacent carbons. Each amino acid can be considered its own spin system, so starting with the NH, correlations are observed to the Hα, Hβ, Hγ, usually to the last H on that side chain. However, no correlations are observed to the next amino acid in the sequence. The amino acids with methyl groups- valine, leucine and isoleucine are easy to assign based upon the presence of the methyl in the TOCSY spectra. Each methyl also has characteristic C13 shift so separating each of those 3 amino acids is straight forward. Lysine and arginine are also readily assigned with the Nitrogen(s) at the end of each of their side chains. The Nitrogen shifts the last CH downfield in both C13 and H1. Also, the NH3+ of lysine is observed and correlates through TOCSY and gCOSY. Glutamine has observable NH2 amide peaks on the end of its side chain making it different that glutamic acid. Aspartic acid and glutamic acid are only different by one carbon, so they are quite similar, but the chemical shifts for aspartate CHβ are slightly different than glutamate. Methionine was difficult due to overlap of its side chain with glutamate and aspartate except for the methyl. Correlations from the methyl in gHMBC which correlates H1 to C13 through multiple bonds and ROESY which correlates through-space connect it to a resonance at 2.34 ppm which connects to the assigned Hα.

The resonances on the staple were identified by TOCSY starting with the alkene peaks at 5.3 ppm (H1)/130 ppm (C13). Ideally there would 4 separate resonances, but only 2 were observed. They can be assigned as the 2 on the same alkene so both staple alkene shifts are the same for the 2 staples. The remainder of the shifts can be observed with TOCSY and gCOSY but were not readily assigned due to overlap. However, the last carbon on the staple side chain can be observed and assigned. Each of the 4 of these is different in carbon shift as observed in gHMBC from a methyl group. This methyl also connects to 4 different carbonyls and 4 carbons with

shifts of about 60 ppm. The 60 ppm carbon is thus the quarternary carbon on the backbone of the peptide that starts the staple side chain. As it is connected to a nitrogen, it is shifted downfield in carbon shift. The carbonyl shift is the backbone amide carbonyl. Thus, the TOCSY connects the alkenes through the staple side chain. The gHMBC connects the methyl to the backbone carbonyl, quarternary carbon, and to the last carbon of the staple side chain.

The last assignments were assigning the NH peaks that are part of the staple. They can be observed in the H1 1D and are singlets, unlike all peptide amides which are doublets except for the side chain NHs which are readily distinguished and the C-terminal amide which is an NH2. As the staple NHs have no connections in H1/H1 through-bond experiments, the through-space ROESY was used to try to connect to other H1 resonances. Correlations are observed in the ROESY to the sequential NH resonances for all 4 NH staple peaks when there was enough resolution from the diagonal. As the 3 sequence specific glutamates and 2 aspartates could not be confidently assigned, a correlation to glutamate could be to any of the 3 in the sequence. The correlations observed are consistent with the structure as drawn.

Predicted molecular weight matches the observed molecular weight for SP9

The predicted molecular weight of SP9 is 2217.6 (calculated from SP9.pdb using Pymol). The molecular weight of the synthesized SP9 as observed by LC/MS is 2222 (see Supplementary Figure 2). Thus, the observed molecular weight is within 0.1% to the predicted molecular weight. This agreement strongly argues that the synthesized SP9 has the correct sequence and that the two staples have formed correctly.

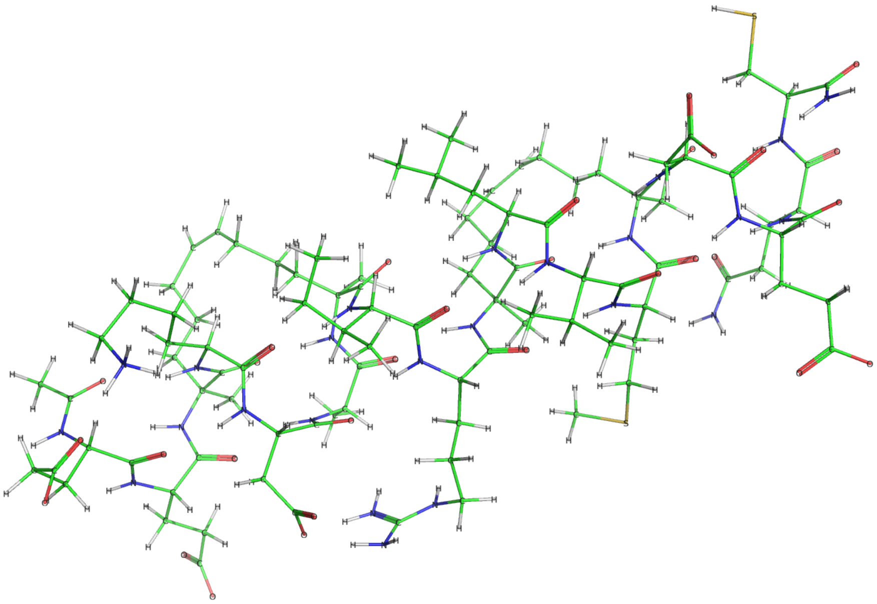

Image of a model of SP9 (the coordinates are available as source data SP9.pdb).

H1 1D

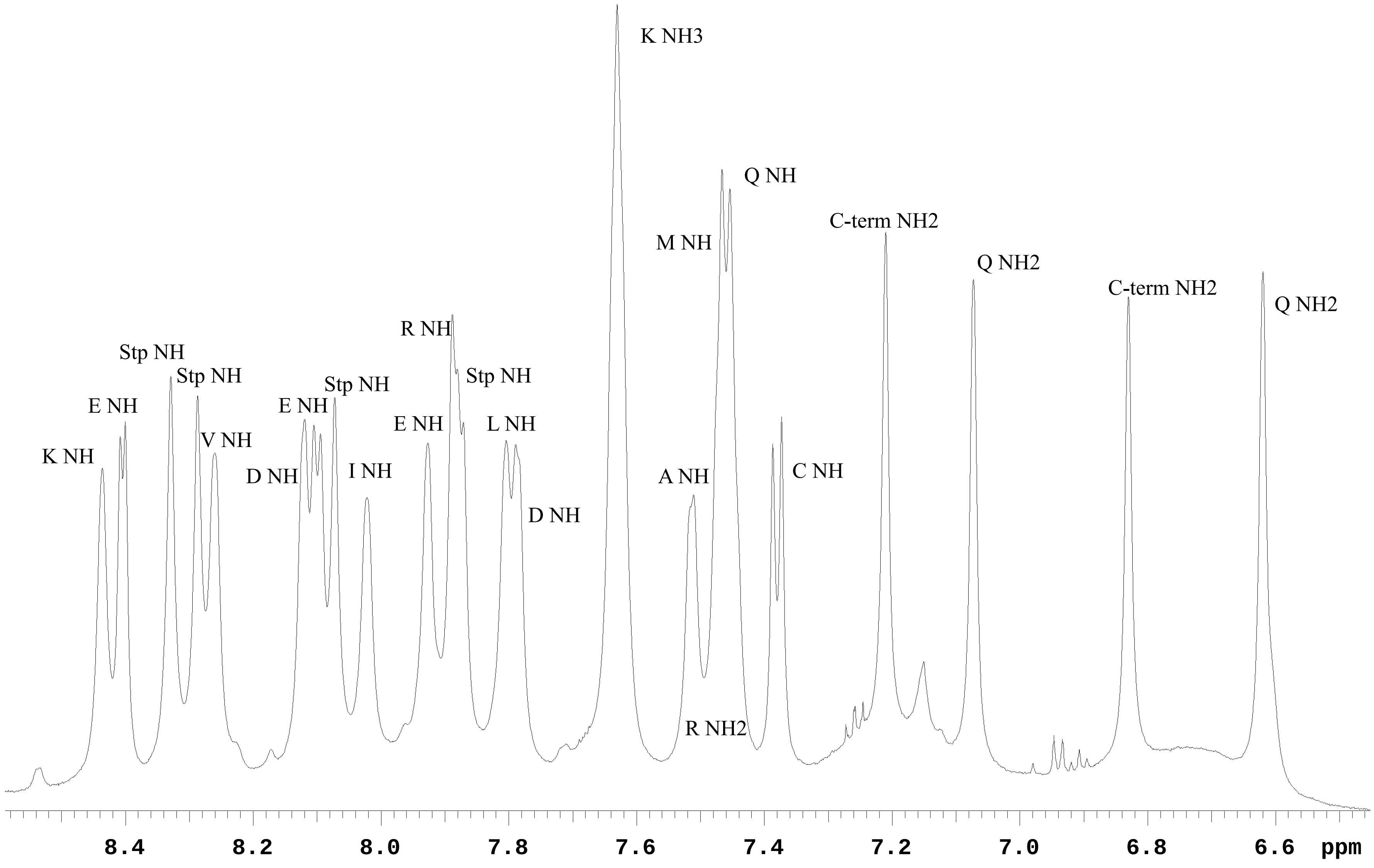

K NH3
Q NH
M NH
C-term NH2
Q NH2
C-term NH2
Q NH2
R NH
Stp NH
Stp NH
Stp NH
Stp NH
E NH
E NH
E NH
K NH
V NH
D NH
I NH
L NH
D NH
A NH
C NH
R NH2
8.4
8.2
8.0
7.8
7.6
7.4
7.2
7.0
6.8
6.6
ppm

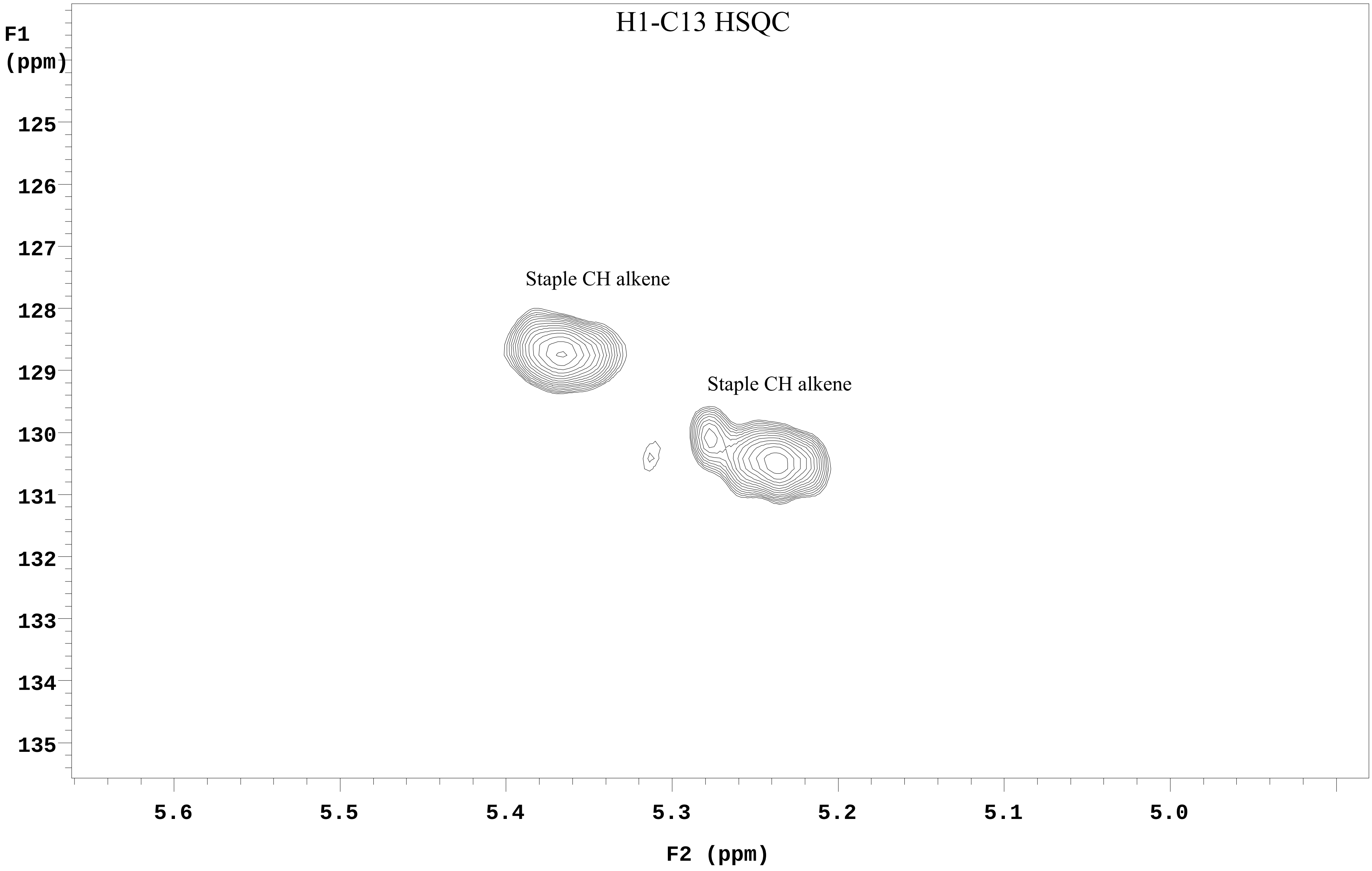
H1-C13 HSQC
F1 (ppm)
125
126
127
128
129
130
131
132
133
134
135
Staple CH alkene
Staple CH alkene
5.6
5.5
5.4
5.3
5.2
5.1
5.0
F2 (ppm)

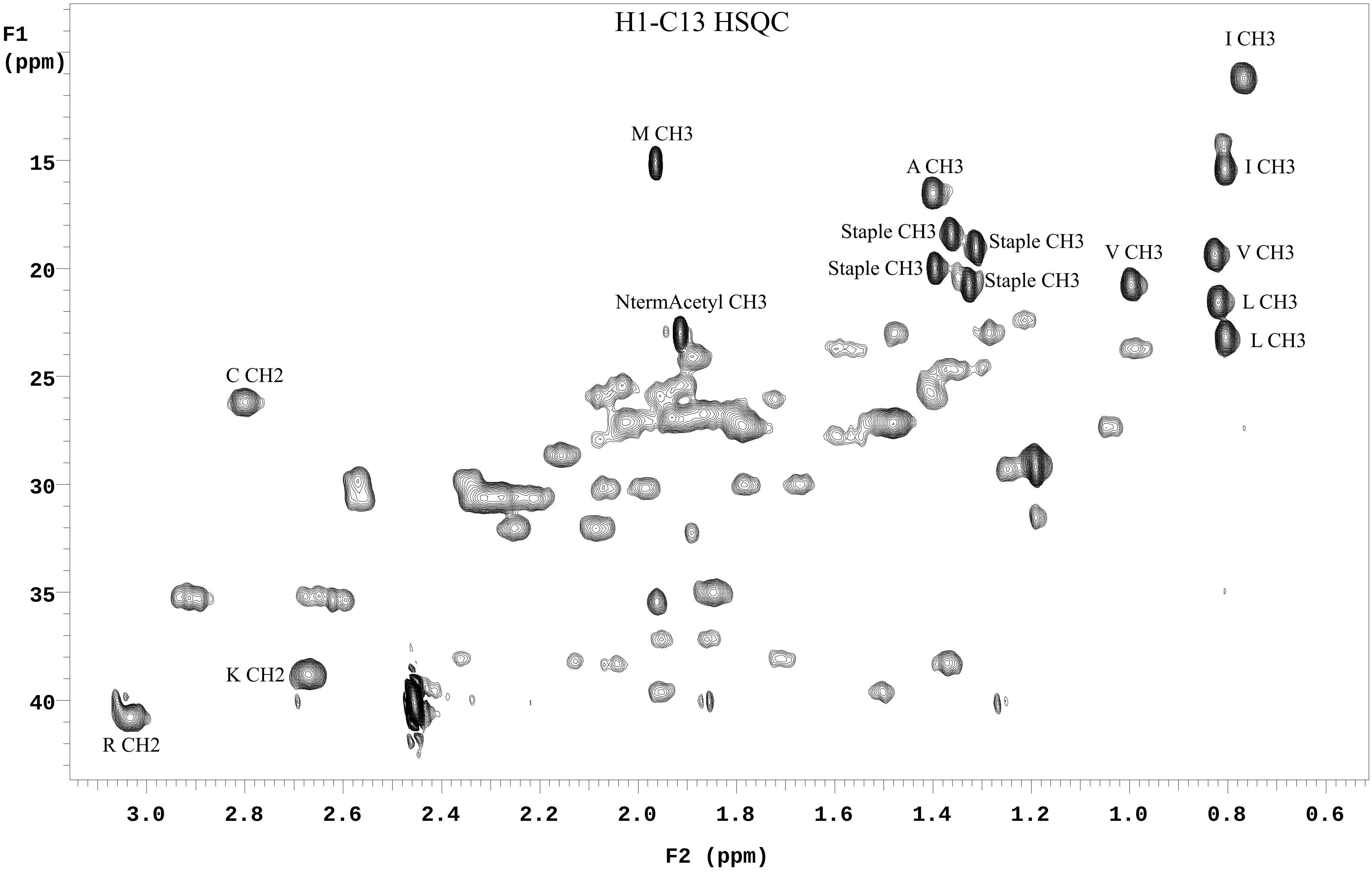

H1-C13 HSQC
F1 (ppm)
F2 (ppm)
I CH3
M CH3
A CH3
I CH3
Staple CH3
Staple CH3
Staple CH3
Staple CH3
V CH3
V CH3
L CH3
L CH3
NtermAcetyl CH3
C CH2
K CH2
R CH2
15
20
25
30
35
40
3.0
2.8
2.6
2.4
2.2
2.0
1.8
1.6
1.4
1.2
1.0
0.8
0.6

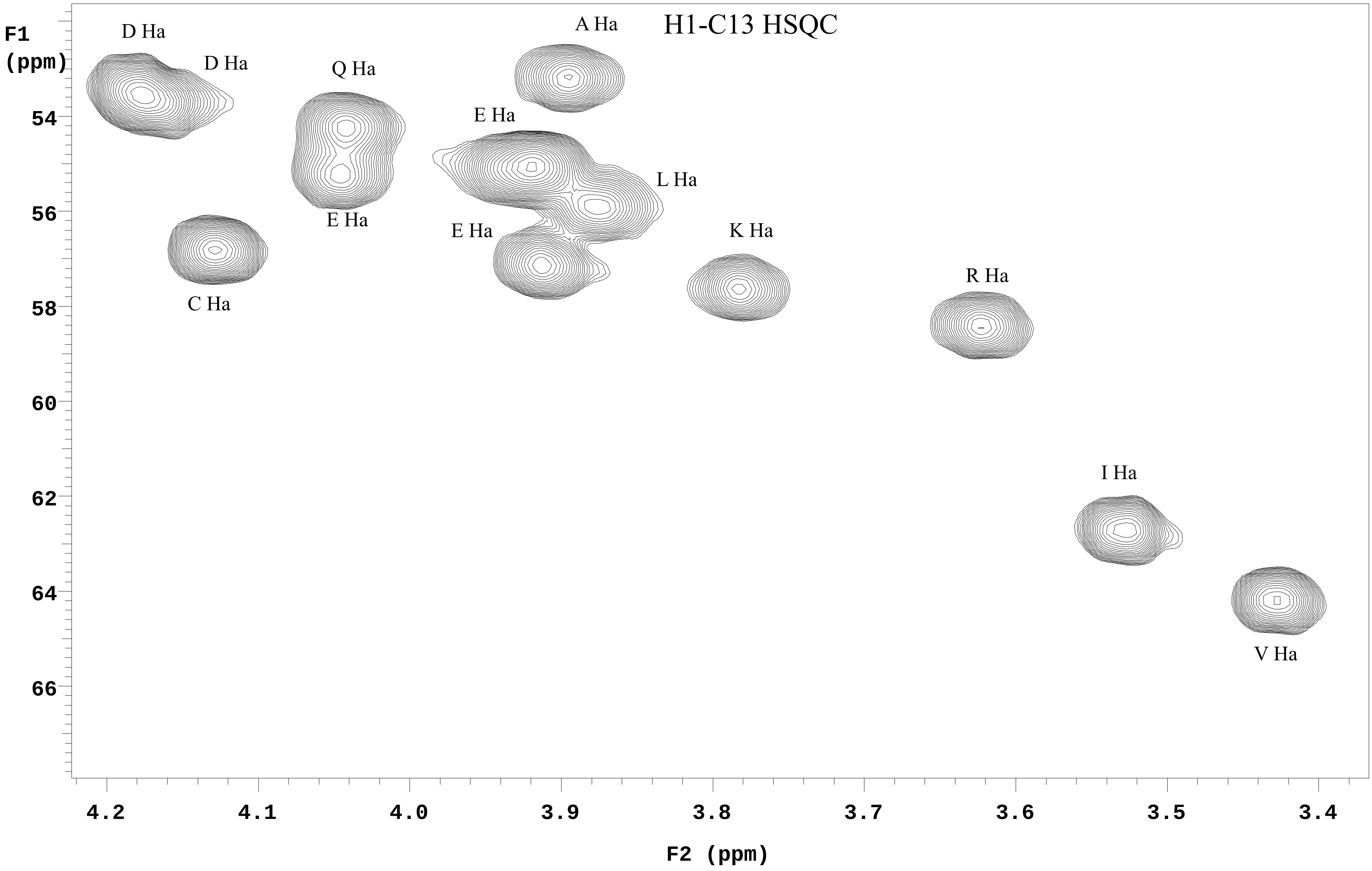

H1-C13 HSQC
F1 (ppm)
F2 (ppm)
D Ha
D Ha
Q Ha
E Ha
A Ha
E Ha
L Ha
E Ha
C Ha
K Ha
R Ha
I Ha
V Ha
54
56
58
60
62
64
66
4.2
4.1
4.0
3.9
3.8
3.7
3.6
3.5
3.4

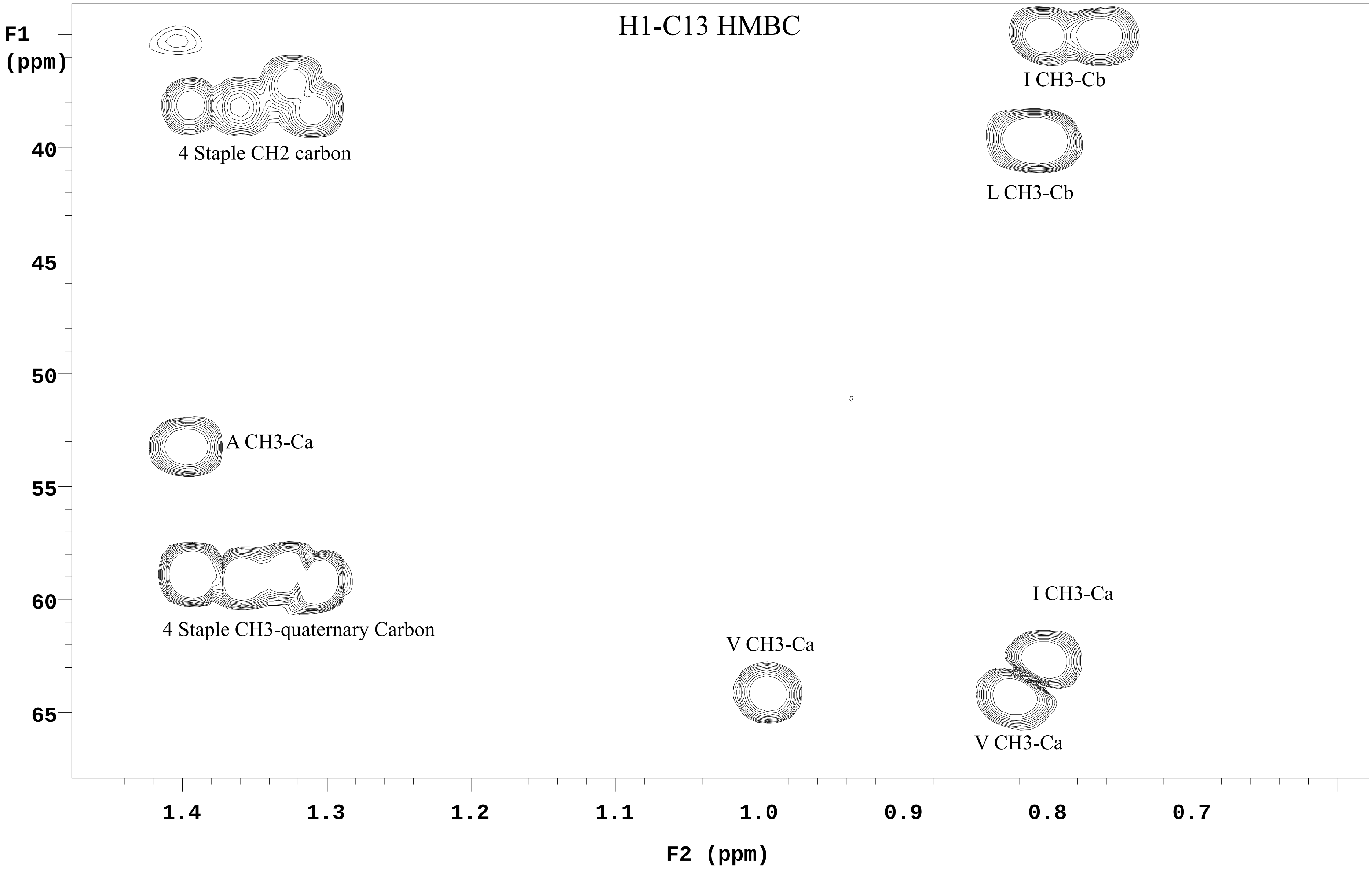
H1-C13 HMBC
F1 (ppm)
40
45
50
55
60
65
4 Staple CH2 carbon
A CH3-Ca
4 Staple CH3-quaternary Carbon
V CH3-Ca
I CH3-Cb
L CH3-Cb
I CH3-Ca
V CH3-Ca
1.4
1.3
1.2
1.1
1.0
0.9
0.8
0.7
F2 (ppm)

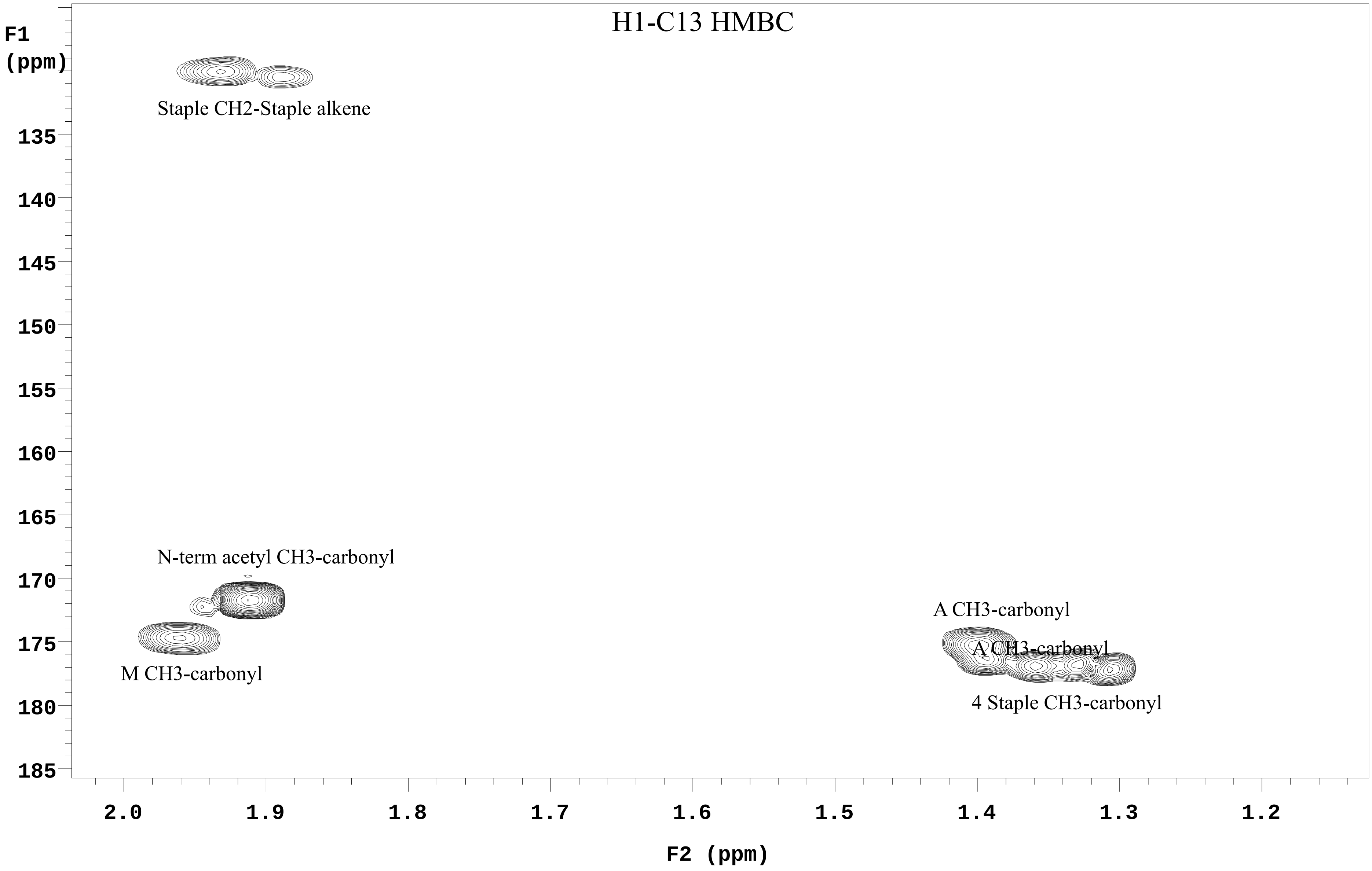
H1-C13 HMBC
F1 (ppm)
135
140
145
150
155
160
165
170
175
180
185
Staple CH2-Staple alkene
N-term acetyl CH3-carbonyl
M CH3-carbonyl
A CH3-carbonyl
A CH3-carbonyl
4 Staple CH3-carbonyl
2.0
1.9
1.8
1.7
1.6
1.5
1.4
1.3
1.2
F2 (ppm)

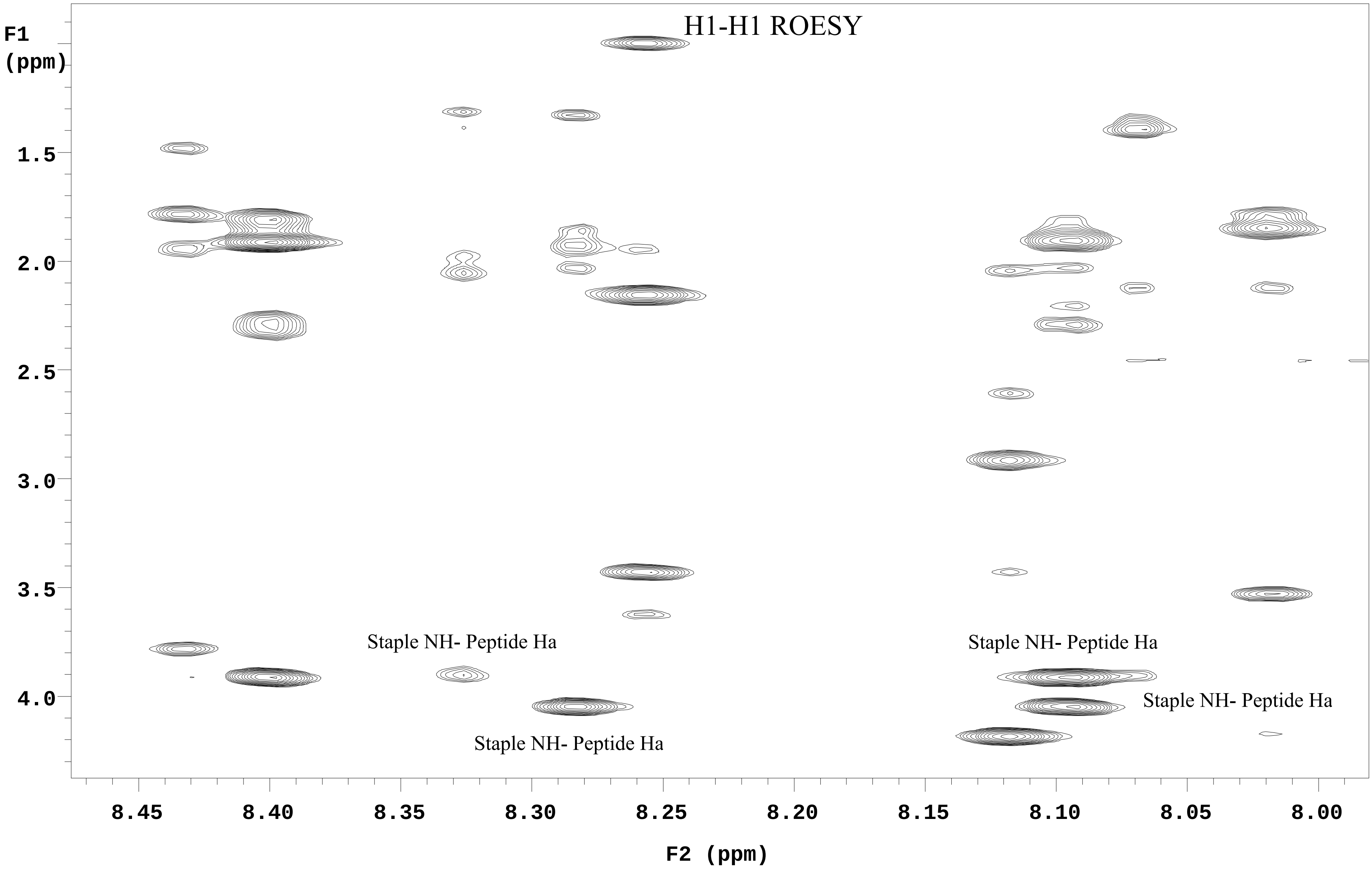
H1-H1 ROESY
F1 (ppm)
1.5
2.0
2.5
3.0
3.5
4.0
Staple NH- Peptide Ha
Staple NH- Peptide Ha
Staple NH- Peptide Ha
Staple NH- Peptide Ha
8.45
8.40
8.35
8.30
8.25
8.20
8.15
8.10
8.05
8.00
F2 (ppm)
