# Supplementary material for: Inhibition of calcium-triggered secretion by hydrocarbon-stapled peptides
Source: Nature. 2022 Mar 23;603(7903):949–56. doi: 10.1038/s41586-022-04543-1 (PMC8967716; doi:10.1038/s41586-022-04543-1)
Supplement: Supplementary file 2 — Reporting Summary [file 41586_2022_4543_MOESM2_ESM.pdf]

nature research

Corresponding author(s): Axel T. Brunger, Ying Lai, Burton F. Dickey, Manfred Frick

Last updated by author(s): Jan. 22, 2022

# Reporting Summary

Nature Research wishes to improve the reproducibility of the work that we publish. This form provides structure for consistency and transparency in reporting. For further information on Nature Research policies, see Authors & Referees and the Editorial Policy Checklist.

## Statistics

For all statistical analyses, confirm that the following items are present in the figure legend, table legend, main text, or Methods section.

| n/a | Confirmed | |
|---|---|---|
| ☐ | ☒ | The exact sample size (*n*) for each experimental group/condition, given as a discrete number and unit of measurement |
| ☐ | ☒ | A statement on whether measurements were taken from distinct samples or whether the same sample was measured repeatedly |
| ☐ | ☒ | The statistical test(s) used AND whether they are one- or two-sided<br>*Only common tests should be described solely by name; describe more complex techniques in the Methods section.* |
| ☐ | ☒ | A description of all covariates tested |
| ☐ | ☒ | A description of any assumptions or corrections, such as tests of normality and adjustment for multiple comparisons |
| ☐ | ☒ | A full description of the statistical parameters including central tendency (e.g. means) or other basic estimates (e.g. regression coefficient) AND variation (e.g. standard deviation) or associated estimates of uncertainty (e.g. confidence intervals) |
| ☐ | ☒ | For null hypothesis testing, the test statistic (e.g. *F*, *t*, *r*) with confidence intervals, effect sizes, degrees of freedom and *P* value noted<br>*Give P values as exact values whenever suitable.* |
| ☒ | ☐ | For Bayesian analysis, information on the choice of priors and Markov chain Monte Carlo settings |
| ☒ | ☐ | For hierarchical and complex designs, identification of the appropriate level for tests and full reporting of outcomes |
| ☒ | ☐ | Estimates of effect sizes (e.g. Cohen's *d*, Pearson's *r*), indicating how they were calculated |

*Our web collection on statistics for biologists contains articles on many of the points above.*

## Software and code

Policy information about availability of computer code

| | |
|---|---|
| Data collection | The HAEC data collection was performed with Leica LAS X v3.1.5.16308, Li-Cor® Odyssey® Fc Imaging System v5.2. The data for the single vesicle fusion experiments and single molecule counting experiments were collected by smCamera developed by Taekjip Ha, Johns Hopkins University, Baltimore. |
| Data analysis | Data analysis was performed for the HAEC experiments with MS Excel for Mac v16.36, GraphPad Prism 7, NIH ImageJ v.2.0.0-rc69. For the mouse experiments, ImagePro 5.1 (Media Cybernetics) was used. For the single vesicle fusion experiments, single molecule counting experiments, fluorescence anisotropy experiments, and circular dichroism experiments, OriginPro 8, Matlab-2021b were used. EMAN2-2.91 was used to analyze the Cryo-EM images in Extended Data Fig. 6a. NAMD2 2.14b1 was used for the molecular dynamics simulations.<br><br>Matlab analysis scripts for the single vesicle fusion, single molecule counting experiments, and smCamera file conversions are available in the Zenodo repository https://doi.org/10.5281/zenodo.637058 . |

For manuscripts utilizing custom algorithms or software that are central to the research but not yet described in published literature, software must be made available to editors/reviewers. We strongly encourage code deposition in a community repository (e.g. GitHub). See the Nature Research guidelines for submitting code & software for further information.

## Data

Policy information about availability of data

All manuscripts must include a data availability statement. This statement should provide the following information, where applicable:

- Accession codes, unique identifiers, or web links for publicly available datasets
- A list of figures that have associated raw data
- A description of any restrictions on data availability

The imaging data for the studies of primary human airway epithelial cells (HAECs), for the mucin secretion and airway mucus occlusion studies in mice, and the NAMD input files and trajectories of the molecular dynamics simulations are available in the Dryad repository doi:10.5061/dryad.dz08kprz7 . Full versions of blots

are provided in Supplementary Fig. 1. Excel spreadsheet files with all data points and analyses are provided as Source Data (Fig1.xlsx, Fig2.xlsx, Fig3.xlsx, Fig4.xlsx, Fig5.xlsx, ED_Fig3.xlsx, ED_Fig4.xlsx, ED_Fig5.xlsx, ED_Fig6.xlsx, ED_Fig7.xlsx, ED_Fig8.xlsx). A model of SP9 is provided as Source Data (SP9.pdb), and endpoints of the molecular dynamics simulations of Syt1 C2B : SP9 and Syt1 C2B : P9 are provided as Source Data (SP9_simulations.pdb, P9_simulations.pdb, respectively).

# Field-specific reporting

Please select the one below that is the best fit for your research. If you are not sure, read the appropriate sections before making your selection.

☒ Life sciences ☐ Behavioural & social sciences ☐ Ecological, evolutionary & environmental sciences

For a reference copy of the document with all sections, see nature.com/documents/nr-reporting-summary-flat.pdf

# Life sciences study design

All studies must disclose on these points even when the disclosure is negative.

| | |
|---|---|
| Sample size | For HAEC experiments peptide and control treatments were conducted within ALI cultures from the same donor. ALI cultures were prepared from 2 donors for peptide internalisation assays and at least 4 different donors for each set of mucin secretion experiments (i.e., different doses, incubation times). 4 donors were considered sufficient to account for donor variation in mucin secretion assays. Donor subject sex, age and smoking status is listed in Supplementary Table 2. Sample size for HAEC experiments was based on previous experience and common practice (this included repeating all experiments in > 3 individual donors for mucin secretion assays). For studies with mice (Fig. 1, 5, Extended Data Fig. 1, 9), two independent experiments were combined to give a total of 10-11 mice per group. For single vesicle fusion experiments (Fig. 3, Extended Data Fig. 4, 5, 7) see Supplementary Table 1 for details about sample size and measurements. |
| Data exclusions | For HAEC experiments, ALI cultures were excluded when ALI was disturbed on day of experiment (i.e. epithelial leakage) and in very rare cases when analysis of dot blot signal was impaired. For single vesicle content mixing experiments, PM vesicles with multiple associated SV, SG, VAMP2, VAMP8 vesicle were excluded. |
| Replication | For HAEC experiments analysing mucin secretion all experiment were conducted in individual ALI filters coming from a miniumum of 4 individual donors, n numbers represent the number of ALI filters. For peptide internalisation experiments in HAEC experiments were repeated twice in ALI cultures from 2 donors. For experiments with mice (Figure 1 and 5), twice. For the single vesicle fusion experiments ((Fig. 3, Extended Data Fig. 4, 5, 7) see Supplementary Table 1 for details for details about repeats. |
| Randomization | For HAEC experiments, donors were selected randomly from our depository. Individual ALI cultures from the same donor were then randomly allocated to control an treatment groups. Control experiments and all experimental conditions were conducted in samples (ALI cultures) from the same donor. Hence, covariates including sex, age, clinical history were identical in all conditions. For animal experiments, mice from appropriate genotypes were randomly assigned to groups for all the conditions. |
| Blinding | For HAEC experiments, none. The entire analysis pipeline to quantify blot intensities was defined upfront (i.e. ROI size and location for detection of signal areas and background) and maintained identically for all analyses. For animal experiments investigators were blinded to the mouse group allocation during data collection and analysis. Also, mouse airway images were analyzed by investigators blinded to animal's genotype and treatment. |

# Reporting for specific materials, systems and methods

We require information from authors about some types of materials, experimental systems and methods used in many studies. Here, indicate whether each material, system or method listed is relevant to your study. If you are not sure if a list item applies to your research, read the appropriate section before selecting a response.

## Materials & experimental systems

| n/a | Involved in the study | |
|---|---|---|
| ☐ | ☒ | Antibodies |
| ☒ | ☐ | Eukaryotic cell lines |
| ☒ | ☐ | Palaeontology |
| ☐ | ☒ | Animals and other organisms |
| ☒ | ☐ | Human research participants |
| ☒ | ☐ | Clinical data |

## Methods

| n/a | Involved in the study | |
|---|---|---|
| ☒ | ☐ | ChIP-seq |
| ☒ | ☐ | Flow cytometry |
| ☒ | ☐ | MRI-based neuroimaging |

## Antibodies

| | |
|---|---|
| Antibodies used | Anti-MUC5AC Mouse Monoclonal Antibody clone 45M1; cat.# MA1-21907, Thermo Scientific (dilution: 1:100 for immunocytochemistry; 1:250 for dot blots)<br>AlexaFluor 488-labelled anti-mouse secondary antibody, cat.# A-11001, Thermo Scientific (dilution 1:500)<br>IRDye® 800CW Donkey anti-Mouse IgG Secondary Antibody, cat.# 926-33212, Li-Cor (dilution 1:10000)<br>IRDye® 680RD Donkey anti-Mouse IgG Secondary Antibody , cat.# 926-68072, Li-Cor(dilution 1:10000) |

| | |
|---|---|
| | Anti-Syt2 rabbit polyclonal antibody ab113545 from Abcam, validated by manufacturer, confirmed by experimentation on the Syt2-deletant tissues<br>Goat Anti-Rabbit (HRP-conjugated) ab205718 from Abcam<br>THE HRP ANTIBODY IS NOT MENTIONED IN THE TEXT, THE ANTI-CCSP ANTIBODY (EXT FIG 9D) IS NOT LISTED HERE |
| Validation | The specificity of the commercially available anti-Muc5AC Antibody from Thermo scientific has been validated in many different publications (available on vendor website) and it has been used on human samples with many different techniques (IF,WB,Elisa). Validation of anti-Mucin and anti-Syt2 antibodies in mice was done using the relevant mouse knockouts. A |

## Animals and other organisms

Policy information about studies involving animals; ARRIVE guidelines recommended for reporting animal research

| | |
|---|---|
| Laboratory animals | Mice:<br>Mutant mice were received on a 129/Sv:C57BL/6 background, as referenced in the manuscript. They were subsequently backcrossed 10 times to C57BL/6J background to match the standard C57BL/6J wild type mice.<br><br>The animals were housed in specific pathogen-free conditions on a 12-hr light/dark cycle with food and water ad libitum. The number of animals used was the minimum that is consistent with scientific integrity and regulatory acceptability, consideration having been given to the welfare of individual animals in terms of the number and extent of procedures to be carried out on each animal. |
| Wild animals | No wild animals were used in this study. |
| Field-collected samples | No field-collected samples were used in this study. |
| Ethics oversight | All the mice work was conducted in accordance with the UT MD Anderson Cancer Center Institutional Animal Care and Use Committee (IACUC) guidelines, and under the IACUC supervision; protocol No 00001214-RN02. |

Note that full information on the approval of the study protocol must also be provided in the manuscript.
