# Supplementary material for: Inhibition of calcium-triggered secretion by hydrocarbon-stapled peptides
Source: Nature. 2022 Mar 23;603(7903):949–56. doi: 10.1038/s41586-022-04543-1 (PMC8967716; doi:10.1038/s41586-022-04543-1)
Supplement: Supplementary file 4 — Data summary table for the mouse airway mucin secretion and mucus occlusion experiments. [file 41586_2022_4543_MOESM4_ESM.docx]

| **Supplementary Table 1. Data summary table for the mouse airway mucin secretion and mucus occlusion experiments**   \| Experimental group \| Independent repeat sets \| Data points n (No of mice) \| No of images analyzed \|  \| \| --- \| --- \| --- \| --- \| --- \| \|  \| \| \| \| \| \| Experiment: Mucin secretion in Syt2 mice (Related to Figure 1b) \| \| \| \| \| \| WT \| 2 \| 10 \| 310 \|  \| \| F/F \| 2 \| 9 \| 279 \|  \| \| D/D \| 2 \| 10 \| 324 \|  \| \|  \| \| \| \| \| \| Experiment: Mucus occlusion in Syt2 mice (Related to Figure 1d) \| \| \| \| \| \| WT \| 2 \| 10 \| 249 \|  \| \| F/F \| 2 \| 10 \| 261 \|  \| \| D/D \| 2 \| 11 \| 282 \|  \| \|  \| \| \| \| \| \| Experiment: Mucin secretion in peptides-treated mice (Related to Figure 5d) \| \| \| \| \| \| PBS \| 2 \| 19 \| 223 \|  \| \| PEN-P9-Cy3 \| 2 \| 10 \| 109 \|  \| \| PEN-SP9-Cy3 \| 2 \| 15 \| 186 \|  \| \|  \| \| \| \| \| \| Experiment: Mucus occlusion in peptides-treated mice (Related to Figure 5f) \| \| \| \| \| \| PBS \| 2 \| 13 \| 168 \|  \| \| PEN-P9-Cy3 \| 2 \| 7 \| 98 \|  \| \| PEN-SP9-Cy3 \| 2 \| 13 \| 194 \|  \| \|  \|  \|  \|  \|  \| \|  \|  \|  \|  \|  \| \| Experimental group \| **Independent repeat sets** \| **Data points n (No of cells)** \| **No of images analyzed** \|  \| \|  \|  \|  \|  \|  \| \| Experiment: Peptides penetration into airway cells (Related to Figure 5b) \| \| \| \| \| \| PBS \| 4 \| N/D \| 18 \|  \| \| PEN-P9-Cy3 \| 4 \| 361 \| 6 \|  \| \| PEN-SP9-Cy3 \| 4 \| 260 \| 6 \|  \| |  |  |  |
| --- | --- | --- | --- | --- | --- | --- | --- | --- | --- | --- | --- | --- | --- | --- | --- | --- | --- | --- | --- | --- | --- | --- | --- | --- | --- | --- | --- | --- | --- | --- | --- | --- | --- | --- | --- | --- | --- | --- | --- | --- | --- | --- | --- | --- | --- | --- | --- | --- | --- | --- | --- | --- | --- | --- | --- | --- | --- | --- | --- | --- | --- | --- | --- | --- | --- | --- | --- | --- | --- | --- | --- | --- | --- | --- | --- | --- | --- | --- | --- | --- | --- | --- | --- | --- | --- | --- | --- | --- | --- | --- | --- | --- | --- | --- | --- | --- | --- | --- | --- | --- | --- | --- | --- | --- | --- | --- | --- | --- | --- | --- | --- | --- | --- | --- | --- | --- | --- | --- | --- | --- | --- | --- | --- | --- | --- | --- | --- | --- | --- | --- | --- | --- | --- | --- | --- | --- | --- | --- | --- | --- | --- | --- | --- | --- | --- | --- | --- | --- |

Each repeat experiment is conducted with several mixed freshly thawed aliquots of reagents, minimizing the lot and pipetting variations.
