# Supplementary material for: Inhibition of calcium-triggered secretion by hydrocarbon-stapled peptides
Source: Nature. 2022 Mar 23;603(7903):949–56. doi: 10.1038/s41586-022-04543-1 (PMC8967716; doi:10.1038/s41586-022-04543-1)
Supplement: Supplementary file 5 — Data summary table for the single-vesicle fusion experiments. [file 41586_2022_4543_MOESM5_ESM.docx]

| **Supplementary Table 2. Data summary table for the single vesicle fusion experiments**   \| Tested peptides or conditions \| Ca^2+^-independent  fusion events \| Ca^2+^-triggered  fusion events \| Number of analyzed  vesicle pairs \| Repeats \| \| --- \| --- \| --- \| --- \| --- \| \| neuronal SNAREs and Syt1 (related to Extended Data Figure 4) \| \| \| \| \| \| None \| 750 \| 846 \| 9557 \| 5 \| \| P0 \| 493 \| 591 \| 6543 \| 3 \| \| SP9 \| 165 \| 272 \| 7122 \| 4 \| \| neuronal SNAREs only (related to Extended Data Figure 5) \| \| \| \| \| \| None \| 410 \|  \| 6980 \| 8 \| \| P0 \| 104 \|  \| 2020 \| 4 \| \| SP9 \| 336 \|  \| 6546 \| 4 \| \| neuronal SNAREs and Syt1_QM (related to Extended Data Figure 5) \| \| \| \| \| \| None \| 320 \| 384 \| 6480 \| 3 \| \| SP9 \| 242 \| 260 \| 2989 \| 3 \| \| airway SNAREs and Syt2 (related to Extended Data Figure 7) \| \| \| \| \| \| None \| 461 \| 657 \| 8377 \| 6 \| \| P0 \| 113 \| 219 \| 2306 \| 3 \| \| SP9 \| 274 \| 250 \| 8445 \| 4 \| \| airway SNAREs only (related to Extended Data Figure 7) \| \| \| \| \| \| None \| 54 \|  \| 2578 \| 6 \| \| P0 \| 38 \|  \| 1448 \| 3 \| \| SP9 \| 22 \|  \| 966 \| 4 \| \| the airway system of complete reconstitution (related to Figure 3) \| \| \| \| \| \| No Munc13-2* \| 18 \| 30 \| 2751 \| 5 \| \| Munc13-2*  (500 µM Ca^2+^) \| 82 \| 647 \| 1863 \| 3 \| \| Munc13-2*+SP9  (500 µM Ca^2+^) \| 41 \| 106 \| 1507 \| 3 \| \| Munc13-2*  (50 µM Ca^2+^) \| 95 \| 715 \| 2102 \| 4 \| \| Munc13-2*+SP9  (50 µM Ca^2+^) \| 23 \| 89 \| 1155 \| 3 \| |  |  |  |
| --- | --- | --- | --- | --- | --- | --- | --- | --- | --- | --- | --- | --- | --- | --- | --- | --- | --- | --- | --- | --- | --- | --- | --- | --- | --- | --- | --- | --- | --- | --- | --- | --- | --- | --- | --- | --- | --- | --- | --- | --- | --- | --- | --- | --- | --- | --- | --- | --- | --- | --- | --- | --- | --- | --- | --- | --- | --- | --- | --- | --- | --- | --- | --- | --- | --- | --- | --- | --- | --- | --- | --- | --- | --- | --- | --- | --- | --- | --- | --- | --- | --- | --- | --- | --- | --- | --- | --- | --- | --- | --- | --- | --- | --- | --- | --- | --- | --- | --- | --- | --- | --- | --- | --- | --- | --- | --- | --- | --- | --- | --- | --- | --- | --- | --- | --- | --- | --- | --- | --- | --- | --- | --- | --- | --- | --- | --- | --- | --- | --- | --- | --- | --- | --- |

Among each repeat experiment there are at least three different protein preps and vesicle reconstitutions, so the variations observed in the bar charts reflect sample variations as well as variations among different flow chambers. For the definition of the repeat experiments see Methods.
