# Supplementary material for: Inhibition of calcium-triggered secretion by hydrocarbon-stapled peptides
Source: Nature. 2022 Mar 23;603(7903):949–56. doi: 10.1038/s41586-022-04543-1 (PMC8967716; doi:10.1038/s41586-022-04543-1)
Supplement: Supplementary file 6 — Donor participant sex, age and smoking status for HAE cell experiments. [file 41586_2022_4543_MOESM6_ESM.docx]

**Supplementary Table 3:** Donor subject sex, age and smoking status for HAEC experiments

| Donor | Age | Sex | Smoking history | related Fig. |
| --- | --- | --- | --- | --- |
| 1 | 59 | m | unknown | Fig 4 |
| 2 | 51 | f | unknown | Fig 4 |
| 3 | 63 | f | unknown | Fig 4 |
| 4 | 55 | m | unknown | Fig 4 |
| 5 | 48 | m | non smoker | ED. Fig 8 |
| 6 | 60 | f | non smoker | ED. Fig 8 |
| 7 | 49 | m | smoker | ED. Fig 8 |
| 8 | 64 | m | unknown | ED. Fig 8 |
| 9 | na | m | non smoker | ED. Fig 8 |
| 10 | 57 | m | unknown | ED. Fig 8 |
| 11 | 31 | m | smoker | ED. Fig 8 |
| 12 | 48 | f | unknown | ED. Fig 8 |
